# Neural Correlates of Huntington’s Disease Based on Electroencephalography (EEG): A Mechanistic Review and Discussion of Excitation and Inhibition (E/I) Imbalance

**DOI:** 10.3390/jcm14145010

**Published:** 2025-07-15

**Authors:** James Chmiel, Jarosław Nadobnik, Szymon Smerdel, Mirela Niedzielska

**Affiliations:** 1 Faculty of Physical Culture and Health, Institute of Physical Culture Sciences, University of Szczecin, Al. Piastów 40B blok 6, 71-065 Szczecin, Poland; 2 School of Sports Championships FASE—Football Academy School of Excellence, Kelvin Grove, QLD 4059, Australia; 3 Department of the History of Medicine and Medical Ethics, Pomeranian Medical University, 70-204 Szczecin, Poland

**Keywords:** Huntington’s disease, EEG, electroencephalography, electroencephalogram, brain oscillations, QEEG, neurophysiology, neural correlates, E/I imbalance

## Abstract

**Introduction**: Huntington’s disease (HD) disrupts cortico-striato-thalamocortical circuits decades before clinical onset. Electroencephalography (EEG) offers millisecond temporal resolution, low cost, and broad accessibility, yet its mechanistic and biomarker potential in HD remains underexplored. We conducted a mechanistic review to synthesize half a century of EEG findings, identify reproducible electrophysiological signatures, and outline translational next steps. **Methods**: Two independent reviewers searched PubMed, Scopus, Google Scholar, ResearchGate, and the Cochrane Library (January 1970–April 2025) using the terms “EEG” OR “electroencephalography” AND “Huntington’s disease”. Clinical trials published in English that reported raw EEG (not ERP-only) in human HD gene carriers were eligible. Abstract/title screening, full-text appraisal, and cross-reference mining yielded 22 studies (~700 HD recordings, ~600 controls). We extracted sample characteristics, acquisition protocols, spectral/connectivity metrics, and neuroclinical correlations. **Results**: Across diverse platforms, a consistent spectral trajectory emerged: (i) presymptomatic carriers show a focal 7–9 Hz (low-alpha) power loss that scales with CAG repeat length; (ii) early-manifest patients exhibit widespread alpha attenuation, delta–theta excess, and a flattened anterior-posterior gradient; (iii) advanced disease is characterized by global slow-wave dominance and low-voltage tracings. Source-resolved studies reveal early alpha hypocoherence and progressive delta/high-beta hypersynchrony, microstate shifts (A/B ↑, C/D ↓), and rising omega complexity. These electrophysiological changes correlate with motor burden, cognitive slowing, sleep fragmentation, and neurovascular uncoupling, and achieve 80–90% diagnostic accuracy in shallow machine-learning pipelines. **Conclusions**: EEG offers a coherent, stage-sensitive window on HD pathophysiology—from early thalamocortical disinhibition to late network fragmentation—and fulfills key biomarker criteria. Translation now depends on large, longitudinal, multi-center cohorts with harmonized high-density protocols, rigorous artifact control, and linkage to clinical milestones. Such infrastructure will enable the qualification of alpha-band restoration, delta-band hypersynchrony, and neurovascular coupling as pharmacodynamic readouts, fostering precision monitoring and network-targeted therapy in Huntington’s disease.

## 1. Introduction

Huntington’s disease (HD) is an autosomal dominant neurodegenerative disorder caused by a pathological expansion of CAG trinucleotide repeats in the huntingtin (HTT) gene on chromosome 4p16.3 [1]. This expansion leads to the production of mutant huntingtin protein with an extended polyglutamine tract, which aggregates and disrupts critical cellular processes such as vesicular transport and transcriptional regulation [2]. The HTT mutation is inherited in an autosomal dominant manner, conferring a 50% risk of transmission to the offspring of an affected parent [3]. The phenomenon of anticipation—further unstable expansion of CAG repeats, especially in the paternal germline—results in earlier disease onset in successive generations [4].

The mean age of HD onset is 35–45 years, and survival after the appearance of motor symptoms typically ranges from 15 to 25 years, with a median of approximately 24 years [5]. Leading causes of death include respiratory complications, infections, and psychiatric factors, including suicide [1]. Key prognostic factors include CAG repeat length (greater expansions correlate with more rapid disease progression) [6], age at onset (juvenile-onset ≤ 20 years is associated with shorter survival) [7], and rate of functional decline, assessed using the Total Functional Capacity (TFC) scale [8]. The global prevalence of HD is approximately 4.88 per 100,000 individuals, with an incidence of 0.48 per 100,000 person-years [9]. Higher rates are reported in Europe and North America compared to Africa and East Asia [9].

Neuropathological features of HD include bilateral striatal atrophy (caudate nucleus and putamen) and degeneration in the cerebral cortex, thalamus, and brainstem [10,11]. Mutant huntingtin undergoes aberrant proteolysis, forms toxic neuronal inclusions, and causes dysregulation of gene transcription essential for neuronal survival [12].

The clinical course of HD encompasses three principal domains, motor, cognitive, and psychiatric [13]. Early stages are characterized by irregular, rapid choreiform movements of the limbs and trunk. As the disease advances, chorea often subsides, giving way to dystonia, muscle rigidity, and bradykinesia [14]. Associated features include gait disturbances, dysarthria, and dysphagia, which increase the risk of aspiration and respiratory infections [15,16,17]. Executive dysfunction—difficulty in planning, cognitive flexibility, and multitasking—often precedes motor symptoms [18,19]. Additional deficits include impaired working memory, slowed information processing, and disorganization of daily activities [20,21]. Common mood disturbances include depression, anxiety, apathy, and irritability. Some patients also exhibit impulsivity, obsessive–compulsive behaviors, and, less frequently, psychotic symptoms such as hallucinations and delusions [22].

Electroencephalography (EEG) is a non-invasive technique for recording the brain’s electrical activity via electrodes placed on the scalp. It measures the summed postsynaptic currents in large populations of cortical neurons [23]. The EEG signal primarily arises from the summation of excitatory and inhibitory synaptic activity of pyramidal neurons, whose dendrites are oriented perpendicular to the skull surface, enabling detection at the microvolt level [24]. The signal is generated by synchronized postsynaptic currents (EPSPs and IPSPs) within these neuronal assemblies. Due to the radial orientation of their dendrites, the resulting electric fields are amplified and summed, allowing detection from cortical regions spanning several square centimeters [25].

Effective signal summation requires the activity of neuronal populations covering at least a few square centimeters of cortex, as the field generated by a single neuron is too weak to be detected on the scalp. EEG recordings are typically conducted using the international 10–20 electrode placement system, which specifies the positioning of between a dozen and several dozen electrodes on the patient’s scalp [26]. Electrodes may be dry or gel-based, and the electrode–skin interface is often optimized with conductive paste [27]. Recordings can be obtained during resting-state conditions (eyes open or closed), while performing cognitive tasks, or during sleep.

One of the main advantages of EEG is its exceptional temporal resolution on the order of milliseconds, making it ideal for tracking rapid neuronal dynamics, such as event-related potentials (ERPs) elicited by sensory, cognitive, or motor events [28]. Other benefits include its non-invasiveness and safety, as the method does not expose the patient to ionizing radiation and is generally well tolerated [23]. EEG is also relatively inexpensive and widely available due to the lower cost of purchasing and maintaining EEG systems compared to more advanced imaging technologies such as magnetic resonance imaging (MRI) or positron emission tomography (PET), making it a commonly used tool in both clinical and research settings [29]. Modern mobile EEG systems are also highly portable, allowing recordings in field settings and naturalistic environments outside of traditional laboratories [30].

Despite its many advantages, EEG also has certain limitations. One is its low spatial resolution, which limits the accurate localization of activity from deep brain structures such as the hippocampus. To address this, advanced source imaging techniques such as Low Resolution Brain Electromagnetic Tomography (LORETA) or beamformer approaches are used [31]. Another major limitation is EEG’s susceptibility to various artifacts that can distort the signal. These artifacts may result from eye movements, muscle activity, or external electromagnetic interference, necessitating the use of appropriate preprocessing, filtering, and artifact removal algorithms [32]. Moreover, EEG has reduced sensitivity to signals from deep brain structures, as these are attenuated before reaching the scalp surface and being detected by electrodes [24].

EEG has a wide range of applications in both clinical diagnostics and research. In epilepsy diagnosis, it enables the detection of epileptiform discharges, and long-term monitoring (including video-EEG) significantly enhances diagnostic sensitivity [33]. In sleep studies, EEG is a core component of polysomnography, enabling the classification of NREM and REM sleep stages and the diagnosis of sleep disorders such as obstructive sleep apnea [34]. In event-related potential (ERP) research, EEG allows for the analysis of time-locked brain responses to stimuli, offering insights into attention, memory, and perceptual mechanisms [24]. EEG also plays a key role in brain–computer interface (BCI) systems, where real-time decoding of mu and beta rhythms enables individuals with motor impairments to control external devices using only their brain activity [35].

HD causes widespread alterations in the nervous system, including the brain. Studies are needed to investigate these changes, elucidate the underlying mechanisms, and identify common correlates and biomarkers linking them to motor, cognitive, and behavioral symptom progression. Such research may enhance our understanding of the disease, allow the identification of “tipping points” associated with symptom deterioration, and, most importantly, support the development of interventions that target these changes and potentially lead to clinical improvement. As mentioned earlier, EEG is a noninvasive, low-cost, and widely used method for diagnosing brain disorders. Despite some limitations, it remains the first-line neuroimaging method routinely used in the diagnosis and study of neurological diseases. In this mechanistic review, we conducted a thorough literature search on EEG in HD using various data mining strategies. Previous findings were analyzed to identify common patterns. Most importantly, a previously unexplored mechanism underlying altered bioelectrical brain activity in HD patients was identified. This review included studies using raw EEG recordings, excluding methodologies such as event-related potentials to maintain homogeneity across included studies. Publications dating from 1970 were considered. Studies from the 1950s and 1960s were excluded, as the continuous evolution of EEG technology means that earlier data may not reliably reflect current measurement standards.

## 2. Methods

The purpose of this mechanistic review is to investigate EEG oscillations associated with Huntington’s disease. Its primary aim is to elucidate the underlying brain correlates and mechanisms identified using EEG, rather than to evaluate the quality of the included studies, although it incorporates certain principles of systematic reviews, such as structured searching and study selection. This review does not follow the full PRISMA procedure typically employed in systematic reviews, due to its emphasis on mechanistic insights. Formal assessment frameworks such as PICOS and Risk of Bias were also excluded.

### 2.1. Data Sources and Search Strategy

To gather relevant studies, four researchers (J.C., J.N., S.S., and M.N.) independently conducted a comprehensive literature search using a combination of keywords related to EEG and Huntington’s disease. The search terms included “EEG” OR “electroencephalography” OR “QEEG” OR “electroencephalogram” AND “Huntington’s disease.” Several databases, including PubMed/Medline, Scopus, ResearchGate, Google Scholar, and Cochrane, were searched in April 2025 with an emphasis on publications released between January 1970 and April 2025. Additionally, the Google browser was used, and the PubMed database was manually searched for related and cited articles.

### 2.2. Study Selection Criteria

To be eligible for inclusion, studies had to be clinical trials with a specific focus on EEG activity in Huntington’s disease and published in English during the specified time period. Articles written in languages other than English, case studies, and review papers were excluded.

### 2.3. Screening Process

A multi-step screening process was employed to identify studies that met the inclusion criteria. Initially, both researchers independently examined the abstracts and titles of all retrieved studies to assess their relevance.

#### 2.3.1. Title and Abstract Screening

To identify studies addressing the use of EEG in Huntington’s disease, each reviewer independently evaluated the titles and abstracts. Only research clearly focused on this topic was considered for the next phase.

#### 2.3.2. Full-Text Assessment

Following the initial screening, the full texts of the remaining articles were reviewed. The primary aim of this assessment was to confirm that each study met the eligibility criteria—specifically, that they were clinical trials involving patients with Huntington’s disease and published in English between January 1970 and April 2025.

## 3. Results

**Figure 1:**
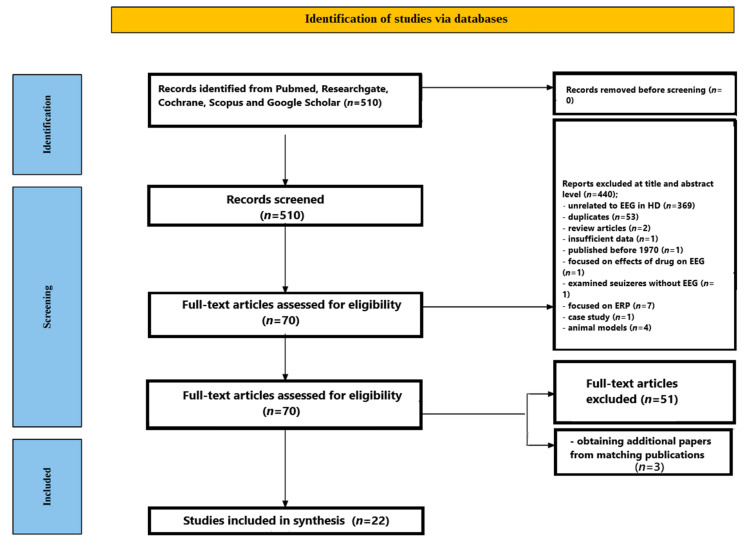
Flow chart depicting the different phases of the systematic review.

The screening process is outlined in Figure 1. A total of 510 studies were identified in the initial database search. After reviewing abstracts and titles, 440 papers were excluded, 369 were unrelated to EEG in Huntington’s disease, 53 were duplicates, and two were review articles. One article was excluded due to insufficient data, one because it predated 1970, one due to its focus on the effects of a drug on EEG in HD, one because it examined seizures without EEG recording, seven for focusing exclusively on ERP, one for being a case study, and four for using animal models. After a full-text evaluation of the remaining 70 papers, 51 were excluded for failing to address EEG in Huntington’s disease. An additional three relevant studies were identified through PubMed’s related article recommendations. In total, 22 studies met all inclusion criteria and were selected for final analysis [36,37,38,39,40,41,42,43,44,45,46,47,48,49,50,51,52,53,54,55,56,57].

### 3.1. Study Types

The included studies are presented in Table 1. Among the included 22 papers, seven partially overlapping experimental designs were identified. The most common was the single-session, cross-sectional case-control format, in which 3- to 10-min eyes-closed EEG recordings from HD gene carriers were compared with age- and sex-matched controls. This classical design appeared in approximately two-thirds of the literature (e.g., studies [37,38,40,42,43,46,47,48,50,51,53,54]) and was used to detect group-level spectral slowing and to relate power or connectivity metrics to clinical scales.

A second group of studies integrated EEG with full-night polysomnography or laboratory-controlled barbiturate sleep to investigate state-dependent abnormalities in spindle generation, K-complexes, and slow-wave dynamics [36,41,52,56].

Third, several teams employed task-based activation paradigms—typically working-memory challenges such as the n-back task—to uncover latent dysfunctions not observable at rest and to measure phase-locked theta or alpha desynchronization/synchronization [45,55].

A more recent strand of research involved simultaneous EEG recordings with other modalities, such as resting-state fMRI [39], functional near-infrared spectroscopy (fNIRS) combined with ECG [53], or a triad of EEG, ECG, and fNIRS [57]. These studies aimed to link cortical oscillations to hemodynamic or autonomic responses and quantify neurovascular coupling efficiency.

Running parallel to these physiological studies is a machine-learning line of research in which high-dimensional spectral or time-domain features are fed into classifiers such as artificial neural networks, support-vector machines or extremely randomized trees to generate diagnostic indices that distinguish HD from control EEGs with accuracies in the 80–90% range (studies [37,43,57]). Many datasets are also explicitly stratified by disease stage, comparing pre-manifest carriers, early-manifest patients, and those with moderate or advanced symptoms to chart how electrophysiological abnormalities expand along the clinical continuum [39,40,44,46,49,50,54]. Only one publication provides genuine longitudinal evidence—twelve patients recorded serially over as much as a decade [42]—yet it shows that once a low-voltage or globally slowed EEG pattern emerges, it tends to persist.

### 3.2. Participant Samples

The 22 eligible articles included nearly 700 individual EEG recordings from HD gene carriers and approximately 600 from matched controls. Study sample sizes ranged from 12 to 105 participants (median ≈ 45). Twenty studies confirmed CAG expansion status through molecular testing (without [41] and [42]). Manifest HD was consistently defined by a diagnostic confidence level (DCL) of 4 on the Unified Huntington’s Disease Rating Scale (UHDRS) or a Total Functional Capacity (TFC) score of ≤13, while pre-manifest carriers had DCL < 2 and UHDRS-TMS ≤ 5. UHDRS was not used in older or purely sleep-lab papers [38,41,42,51].

Approximately one-quarter of patient recordings (≈180 cases, from studies [36,37,39,43,49,50,53,54,55]) were from pre-manifest mutation carriers in their late twenties to early forties (mean age range: 30–44 years). About one-third (≈230 recordings) represented early-manifest or Stage 1–2 HD, generally in the fourth and fifth decades of life (mean 40–52 years; e.g., studies [39,40,43,46,49,51,52,54,55]). The remaining recordings came from moderate to advanced stages—including Stage 3–4 cohorts [40,41,42,47,48,52,56] and a severely choreatic follow-up subgroup [53]—with mean ages clustering around the mid-fifties. The oldest cohort (mean age 57) was the sleep-laboratory sample [52]. The overall sex distribution was balanced (≈52% female), with no systematic bias across disease stages.

All studies used age- and sex-matched healthy control groups; two multimodal investigations [53,57] maintained separate control cohorts for their pre-symptomatic and symptomatic arms to avoid age-related confounding. The aggregate control population ranged from 18 to 82 years, mirroring the HD sample. One study [38] also included an elderly non-HD dementia group (DAT, mean age 77.7 years) to benchmark HD-related slowing against Alzheimer-type cortical dysfunction.

Seven studies [40,46,47,48,51,53,54] analyzed drug-free subgroups (≈120 mutation carriers total) to isolate intrinsic disease effects from dopaminergic or sedative influences. The remaining studies either excluded benzodiazepine users or included medication load as a covariate. Even in mixed samples, detailed tables reported neuroleptic, antidepressant, and anticonvulsant use, allowing for sensitivity analyses.

Twelve studies [39,40,43,44,45,46,48,49,50,52,53,54] administered the UHDRS motor scale; 16 included at least one cognitive measure (MMSE, SDMT, Stroop, or Verbal Fluency), and nine used mood assessment instruments such as the BDI-II or HADS. For pre-manifest participants, estimated time-to-onset—calculated from age and CAG repeat length—was reported in eight studies, with mean CAP or DBS values indicating an average of 10–15 years before expected clinical conversion.

Sleep studies [36,41,52,56] required participants to be suitable for overnight polysomnography (PSG), excluding individuals with severe chorea, where necessary. Multimodal EEG–fMRI or EEG–fNIRS studies [39,53,57] screened participants for MRI compatibility and cardiorespiratory stability. Finally, functional-capacity strata (TFC 0–6 “severe”, 7–10 “moderate”, 11–13 “mild”) were applied in studies [47,49,54] to anchor electrophysiological findings along a clinical severity gradient.

### 3.3. Methodological Approaches

Although the included papers span four decades, their technical workflows form a recognizably coherent pipeline that has gradually become more sophisticated. All studies began with standardized resting- or sleep-EEG acquisition, typically conducted with eyes closed and during mid-morning sessions to minimize circadian variability. Earlier studies employed 8-, 16- or 21-channel 10–20 montages, sampled at 100–256 Hz and recorded in short 3–5 min blocks (e.g., Refs. [37,38,40,41,42,46,47,51]). More recent investigations adopted 32- or 61-channel caps [49], and in two instances, high-density systems with 128 electrodes sampled at 1 kHz [55], enabling source localization and graph-theoretical analyses. Vigilance was monitored in real time and maintained, where necessary, through auditory prompts or by excluding micro-drowsy epochs. Sleep studies combined the scalp EEG array with full polysomnography and occasionally included thiopentone or quinalbarbitone induction to probe spindle physiology [36,41].

Pre-processing methods evolved from visual artifact rejection and simple epoch segmentation (typically ten 4- or 5-s segments per channel) to semi-automated pipelines. These regression-based ocular corrections were followed by manual auditing [46], independent component analysis (ICA) using EEGLAB [49], the fully automated RELAX pipeline for high-density data [54], and strict channel-level rejection of myogenic bursts in choreatic patients. Later studies re-referenced recordings to linked mastoids or an average reference and limited spectral analysis to the 0.5–35 Hz range to match clinical bandwidths. Differences in sampling rates were addressed through resampling or the use of integer-length FFT blocks.

Spectral estimation was consistently conducted using the Fast Fourier transform (FFT), although the granularity of analysis became more refined over time. Early papers quantified the four classical bands—delta, theta, alpha, beta—reporting values as absolute (μV^2^ Hz^−1^) and relative (%) power, sometimes supplemented by composite metrics such as “% EEG Power” (alpha/[alpha + theta]) [38]. Later studies applied log-ratio transformations to stabilize variance [39,46], analyzed 1-Hz bins between 4 and 13 Hz to focus on the theta–alpha boundary [50], or separated periodic from aperiodic 1/f activity using eBOSC before computing a rhythmic signal-to-noise ratio [54]. Peak-frequency and centroid (center-of-gravity) measures were also commonly reported [46,51].

Source analysis and network modeling became central components of recent studies. Low-resolution electromagnetic tomography (LORETA/eLORETA) was routinely applied to each clean epoch to reconstruct intracortical current density on a 5–7 mm grid [39,40,44,48,52,55]. Functional connectivity between these sources was then assessed using the following metrics that reduce volume-conduction artifacts: lagged phase synchronization (LPS) for low-density arrays [44,49], weighted phase-lag index (wPLI) for high-density recordings [54], and EEG–fNIRS phase coherence at shared frequencies (~0.1 Hz) for studies on neurovascular coupling [53]. One study integrated eLORETA-derived alpha power into seed-based resting-state fMRI graphs via psychophysiological interaction modeling [39], effectively linking the electrophysiological and hemodynamic data. Microstate segmentation, another network-level tool, partitioned resting-state EEG into four canonical topographies and analyzed their coverage, frequency, and duration in relation to clinical metrics [48].

Machine learning pipelines extended classical statistical approaches. In study [37], a three-layer backpropagation neural network was trained on 16-channel alpha, theta, and delta spectra, achieving 85% classification accuracy. A subsequent study extracted over 17,000 spectral–topographical features, reduced them using a genetic algorithm, and classified the remainder using a support vector machine [43]. The most recent multimodal approach concatenated 948 time- and wavelet-domain features from EEG, ECG, and fNIRS, and applied an ensemble of extremely randomized trees, yielding 91% cross-validated accuracy [56]. All three studies used patient-wise k-fold validation to prevent information leakage and reported performance using ROC-AUC, precision, and recall metrics. Notably, correlation with UHDRS and SDMT scores indicated that only feature-level composites—not the overall disease-probability indices—tracked clinical variability [43].

Statistical inference followed a consistent hierarchical structure. At the univariate level, repeated-measures ANOVA or mixed-effects models were employed to test band × group interactions, with post-hoc corrections using Duncan or Bonferroni procedures. Multi-electrode topographic comparisons were conducted using t-tests corrected by permutation-based omnibus binomial testing [46], false discovery rate (FDR) across regions of interest (ROI) [39,49], or non-parametric cluster permutation methods within eLORETA space [40,52]. Spectral power and connectivity metrics were regressed against CAG repeat length, disease burden score (DBS), UHDRS-TMS, TFC scores, and neuropsychological *z*-scores, using Spearman or Pearson correlations with FDR correction. Medication status was handled either by excluding affected participants, including it as a covariate in ANCOVA models, or analyzing drug-free subgroups that replicated primary results [40,46,51].

Task-based studies introduced temporal resolution into the analysis. In one working memory paradigm [45], alpha and theta event-related desynchronization/synchronization ERD/ERS were averaged over 15-s encoding intervals, revealing excessive alpha suppression in pre-manifest HD carriers. In the 128-channel n-back experiment [55], the update, maintenance, and readout phases were segmented and analyzed using Morlet-wavelet time–frequency decompositions at the source level, revealing a selective loss of theta synchronization during the update and readout phases only.

### 3.4. Data Analysis and Interpretation

Having outlined the acquisition pipelines, we next examine how different statistical approaches shaped the biological interpretation.

#### 3.4.1. Univariate Statistics and Effect Quantification

Traditional case-control comparisons relied on *t*-tests or one-way ANOVA applied to absolute or relative band-power metrics. Examples include the parietooccipital %EEG-Power ratio that separated both DAT and HD from controls [38], and a binomial omnibus test that deemed 684 electrode-by-band contrasts significant when ≥13 reached *p* < 0.01 after log transformation [46]. More recent studies reported standardized effect sizes: low-alpha suppression in pre-manifest carriers reached Hedge’s *g* = 0.76–0.91, increasing to *g* = 1.67 in early-manifest patients [39], while 1-Hz sub-band deficits around 8 Hz yielded *g* ≈ 0.6 in pre-HD individuals [50]. Confidence intervals were rarely reported, but where available, they supported large and non-overlapping group differences.

#### 3.4.2. Multivariate and Dimension-Reduction Tactics

To mitigate “voxel-count inflation”, several studies employed data reduction before hypothesis testing. Principal component analysis condensed 117 polysomnographic variables into two latent sleep factors, which were then correlated with disease burden scores (DBS) [36]. In machine-learning pipelines, genetic algorithms or recursive feature elimination distilled >17,000 spectral descriptors to ≤30 salient predictors, thereby minimizing collinearity prior to classification using support vector or tree-based models [43,57].

#### 3.4.3. Spatial Statistics and Multiple-Comparison Control

Source-space studies employed non-parametric permutation testing with threshold-free cluster enhancement or randomization-based approaches to maintain family-wise error rates below 0.05 across 2394 voxels [40,52]. Connectivity matrices derived from lagged phase synchronization underwent false discovery rate (FDR) correction (*q* < 0.05), revealing delta-band hypersynchrony that remained significant after controlling for 1860 edge-wise comparisons [49]. These spatial measures were not merely descriptive; reduced global lagged connectivity predicted omega complexity, reflecting cortical network fragmentation in manifest HD [48].

#### 3.4.4. Linking EEG Metrics to Genotype and Phenotype

Correlational analyses consistently associated electrophysiological slowing with genetic load. Narrow-band power gradients (7–8 Hz minus 4–5 Hz) accounted for up to 30% of the variance in CAG repeat length (r = −0.60) and DBS (r = −0.57) in pre-manifest carriers [50]. Prefrontal beta-1 hypersynchrony inversely correlated with Symbol-Digit Modality Test scores (r = −0.81), even in carriers without motor signs [49]. In an EEG–fNIRS study, mixed-effects regression demonstrated that reduced alpha-band neurovascular coherence independently predicted symptomatic status, after adjusting for age and medication [53]. IN sleep studies, caudate-specific atrophy (rather than global cortical loss) accounted for 45% of the variance in slow-wave sleep reduction (ρ = 0.67) [56].

#### 3.4.5. Machine-Learning Inference

Artificial neural networks trained on alpha suppression combined with theta/delta augmentation achieved 85% classification accuracy and generated continuous “abnormality scores” that negatively correlated with estimated years-to-onset (Spearman ρ = −0.71) [37]. An extremely randomized trees ensemble integrating EEG, ECG, and fNIRS data reached 91% accuracy (AUC = 0.96) and, through Gini importance ranking, identified posterior-temporal alpha loss as the most diagnostic feature [57]. Notably, global classifier indices showed poor sensitivity to clinical scales, but secondary feature subsets (Index-A, Index-B) strongly correlated with processing speed (r = 0.86) and motor impairment (r = 0.84), respectively, highlighting the importance of task-specific endpoints [43].

### 3.5. Key Observed EEG Abnormalities

#### 3.5.1. Spectral Power: A Shift from Low-Alpha Loss to Slow-Wave Excess

The earliest electrophysiological signature of HD appears not within the classical delta or theta bands, but at the low-alpha boundary (7–9 Hz)**.** Two independent resting-state studies analyzing 1-Hz sub-bands demonstrated that symptom-free gene carriers already exhibit a selective power reduction in the 7–9 Hz range—yielding medium effect sizes in pre-manifest individuals (Hedge’s *g* ≈ 0.7) and large effect sizes in early-manifest patients (up to *g* = 1.7) [39,50]. This focal deficiency correlates inversely with CAG repeat length and DBS, indicating a direct relationship with toxic gene dosage [39,50,53].

As clinical symptoms develop, the narrow low-alpha dip expands into a widespread alpha suppression (8–12 Hz) observable across the entire scalp. Quantitative EEG mapping and LORETA source analysis consistently indicate that this reduction is most prominent in frontal regions and striato-thalamocortical projection zones [40,46,51]. Alpha centroid values become unstable and display increased variance, suggesting fragmentation of the underlying neural generator networks [46]. Functionally, diminished alpha power is associated with elevated UHDRS motor scores and poorer performance on processing speed and Stroop tasks, with correlation coefficients ranging from 0.4 to 0.6 [46,51,54].

Parallel to the collapse of alpha rhythms, there is a progressive excess of slow-wave activity. In mild-to-moderate manifest HD, absolute delta and theta power increases 30–70% above control means, initially in frontal regions and later spreading posteriorly, while beta power decreases [38,42,47,52]. Electrode-independent centroids for the combined δ/θ spectrum shift toward lower frequencies, and the typical anterior–posterior gradient (frontally dominant delta, and posterior dominant alpha) flattens or reverses [46,47,51]. The dominance of slow-wave activity correlates with both motor impairment and cognitive decline, underscoring its clinical significance [40,46].

State-dependent recordings reinforce this hierarchy. During non-REM sleep, HD patients replace the waking pattern of frontal delta surplus with an alpha surge in the motor cortex, likely reflecting failed cortical inhibition. REM sleep is marked by a bilateral theta–alpha deficit in limbic and posterior association areas, consistent with reported memory and affective disturbances [52]. Earlier barbiturate-induced sleep studies had already noted reduced spindle and K-complex activity, along with low-voltage delta that failed to reach normal amplitude, pointing to a fundamental thalamocortical dysregulation [41,56].

Taken together, the data chart a spectral continuum: an isolated 7–9 Hz power loss in asymptomatic gene carriers evolves into pan-cortical alpha attenuation and eventually into slow-wave dominance and centroid slowing as the disease progresses. This orderly migration of power from higher to lower frequencies provides a straightforward and quantifiable indicator of the progressive disintegration of corticostriatal networks in HD.

#### 3.5.2. Correlations Between EEG Patterns and the Clinical, Genetic, and Functional State of HD

A remarkably consistent set of relationships links the electrophysiological profile of HD with its genetic burden and phenotypic manifestations. The earliest detectable signal—emerging years before overt motor symptoms—is a localized reduction in power at the low-alpha/upper-theta border (7–9 Hz). In two independent resting-state cohorts, this focal reduction scaled inversely with both CAG-repeat length and the composite DBS. A simple gradient between 7–8 Hz and 4–5 Hz power predicted the estimated time to onset [38,50]. Neural network outputs in an early machine-learning study mirrored this trend, becoming progressively more “HD-like” as the predicted onset approached [37].

With the appearance of motor symptoms, the spectral profile expands to include widespread alpha attenuation and excess delta–theta activity. In three large-scale mapping studies, lower alpha and higher slow-wave power consistently correlated with worse performance on the UHDRS motor section. The most pronounced regional effects were located in frontal and limbic areas [40,46,51]. Cognitive correlates followed a similar gradient: reduced alpha power or connectivity in frontoparietal networks predicted slower Symbol–Digit Modalities Test (SDMT) scores, increased Stroop interference, and diminished verbal fluency. In some cases, single features or composite indices accounted for up to 75% of the variance in processing speed [39,43,46,50,54].

Task-based EEG further refines this understanding. Pre-manifest carriers already fail to exhibit normal theta synchronization during the update and readout phases of an n-back working memory task, with the severity of this deficit increasing in line with the CAP (cumulative pathological) score [55].

Network-level analyses add anatomical specificity to these scalar findings. Precise LORETA and high-density scalp analyses show that alpha power is reduced in frontostriatal projection areas, while delta-band hypersynchrony is prominent in bilateral premotor and sensorimotor cortices. The strength of this low-frequency coupling rises with both motor severity and body-mass changes, and within limbic–prefrontal circuits, it correlates with executive dysfunction [44,49]. Microstate segmentation further reveals temporal reorganization: increased expression of microstates A and B, along with diminished expression of microstates C and D, track higher UHDRS scores and lower Mini-Mental State Examination (MMSE) performance. In parallel, rising omega complexity signals progressive cortical fragmentation [48]. Even the neurovascular interface is affected; EEG–fNIRS studies show that alpha-band neurovascular phase coherence is already diminished in presymptomatic carriers and declines further with worsening chorea, suggesting that vascular inefficiency progresses in tandem with network degradation [53].

These correlations extend into sleep and neuroanatomy. Among asymptomatic carriers, greater sleep fragmentation and reduced REM-theta power directly correlate with disease burden scores and subtle cognitive-affective changes [36]. In symptomatic patients, the depth of slow-wave sleep loss correlates not with global cortical atrophy, but specifically with caudate degeneration, reinforcing the basal ganglia’s role in sleep regulation [56]. Finally, the classical “low-voltage” EEG phenotype—defined by the absence of rhythms exceeding 10 μV—emerges early in a subset of patients, persists for years, and is associated with combined frontal-cortical and striatal atrophy, as well as rapid functional decline [42].

Taken together, these converging lines of evidence delineate a coherent pathological continuum: genetic load initially disrupts low-alpha oscillations; subsequent striato-thalamocortical damage drives a global shift toward slow frequencies, accompanied by maladaptive low-frequency hyperconnectivity and fragmentation of faster rhythms. In advanced stages, global voltage suppression and sleep-state disorganization herald terminal functional collapse.

#### 3.5.3. Voltage Amplitude and Topography

The impact of the HTT mutation on EEG amplitude is strikingly consistent: rhythms initially diminish in focal posterior leads and subsequently collapse across the scalp, with some patients ultimately exhibiting near-isoelectric tracings.

Both historical and modern studies converge on two pragmatic thresholds. Using the classical “positive EEG” criterion—defined as the absence of rhythmic activity exceeding 10 μV—Scott et al.’s barbiturate-induced sleep study found that 10 out of 16 symptomatic patients fell below this limit, with two exhibiting near-flat activity (<5 μV); both patients died within a month [42]. A milder amplitude threshold (global peaks < 25 μV) identified 11 out of 16 mild-to-moderate HD cases in a power-spectral study. This low-amplitude subgroup demonstrated significantly poorer motor (QNE) and executive function scores than higher-voltage peers [51].

Amplitude loss is not uniform but follows a characteristic rostro-caudal trajectory.

In healthy adults, alpha amplitudes typically reach 30–50 μV at parieto-occipital sites. Among pre-manifest carriers, alpha is already reduced by 15–20% at O1/O2 electrodes (mean reduction ≈ 3 μV), and by the early-manifest stage, amplitudes have declined by 35–40% across the entire scalp, with maxima over mid-frontal and parietal regions [39,46]. The anterior-posterior (AP) gradient characteristic of normal alpha activity is flattened or even reversed; quantitative EEG confirmed a five-fold attenuation of the alpha-AP gradient in both pre-manifest and drug-free manifest cohorts [47].

LORETA mapping localizes the earliest delta/theta augmentation to the right orbitofrontal cortex and anterior cingulate gyrus [40]. eLORETA analyses reproduce this pattern in bilateral primary motor areas during wakefulness and in the posterior cingulate during REM sleep [52]. In advanced disease, these slow waves reach amplitudes of 50–70 μV, and their voxel-wise power correlates positively with motor severity on the UHDRS-TMS.

Although overall beta power is suppressed, a compensatory “hot-spot” emerges in the left temporal cortex, where peak beta frequency increases from 15.8 Hz (controls) to 17.4 Hz, and local amplitude rises by approximately 30%. This feature correlates with perseverative errors on the Wisconsin Card Sorting Test [51].

As voltage amplitude declines, physiological hallmarks disappear in a sequential fashion: first occipital alpha blocking, followed by photic driving responses, and finally auditory-evoked alpha. By the time low-voltage criteria are met, sleep spindles and K-complexes are absent, even under barbiturate sedation—highlighting profound thalamocortical disengagement [41].

#### 3.5.4. Functional Connectivity and Network Organization

The earliest network-level abnormality detectable via EEG in Huntington’s disease is a selective loss of long-range alpha synchrony, which emerges during the pre-manifest stage. This is followed by an apparently paradoxical increase in low-frequency coupling—primarily in the delta and low-beta ranges—once motor symptoms appear. In the largest connectivity study to date, employing exact-LORETA lagged phase synchronization (LPS) across sixty-one electrodes in 105 participants, mean whole-brain alpha-band LPS in pre-manifest carriers was already 12% lower than in controls, despite normal power. In manifest HD, alpha synchrony decreased by a further 23% [49]. This alpha hypocoherence coincided with an 18% increase in global omega complexity (a measure of spatial independence of sources) that correlated with Shoulson stage [48], suggesting progressive fragmentation of cortical assemblies as the disease advances.

Amid this alpha desynchronization, frequency-specific hypersynchrony patterns emerge. Using the same LORETA-LPS method, manifest patients displayed widespread increases in delta, alpha-1, alpha-2, beta-2, and beta-3 coupling, all surviving FDR correction across 1860 region pairs. The strongest effect was observed between the right prefrontal cortex (Brodmann areas 46/47) and bilateral premotor regions (mean LPS +0.18) [49]. A complementary eLORETA study focused on the sensorimotor network showed that even pre-manifest carriers exhibited heightened delta connectivity between left and right premotor cortices during quiet wakefulness (median LPS 0.44 vs. 0.29). This interhemispheric hypersynchrony intensified across NREM and REM sleep, expanding to somatosensory–motor connections, and extended into theta and alpha bands during REM [44]. Within-group analyses showed that these abnormal couplings were phenotypically relevant: alpha-band REM connectivity between bilateral S1 cortices positively correlated with both UHDRS motor scores and CAG-derived disease burden [44].

Phase-lag measures derived from surface electrodes confirmed the LORETA findings. In an eyes-open resting-state recording processed using the RELAX pipeline, the weighted Phase-Lag Index (wPLI) revealed a diffuse network comprising 76 supra-threshold links (Network-Based Statistic) across 40 electrodes in the delta range (1–4 Hz) in late pre-manifest and early-manifest HD. No theta or alpha wPLI changes survived correction, underlining the band-specific nature of this hyperconnectivity [54]. Behaviorally, stronger delta coupling predicted slower processing speed on the Symbol-Digit Modalities Test, while reduced alpha wPLI was associated with lower Montreal Cognitive Assessment scores after controlling for age.

Temporal microstate analysis provides converging evidence for altered network dynamics. HD patients spent a greater proportion of time in microstate class A (mean coverage: 35.4% vs. 26.8%) and class B, but significantly less in microstates C and D (coverage reductions of 6.2% and 4.9%, respectively) [48]. Since microstate C is believed to index posterior default-mode processing and microstate D frontoparietal control, the shift toward classes A/B likely reflects the over-engagement of sensorimotor and salience networks at the expense of higher-order integrative processes. An increased occurrence of class A correlated with worse motor scores, while shorter class C duration tracked poorer executive performance on the Stroop interference task.

Multimodal studies support and extend the EEG-derived network findings. In a simultaneous EEG–fMRI study, loss of 8–9 Hz power explained 29% of the variance in frontostriatal resting-state hyperconnectivity, implicating a common disinhibitory mechanism linking cortical slowing to circuit-level over-coupling [39]. Separately, EEG–fNIRS recordings demonstrated that phase coherence between alpha rhythms and myogenic (~0.1 Hz) hemodynamic oscillations was already 17% lower in presymptomatic carriers and fell to 32% below control levels in manifest HD, indicating an early and progressive breakdown in neurovascular synchrony [53].

Taken together, these findings delineate a two-stage trajectory of network reorganization. Stage 1 (pre-manifest): diffuse attenuation of alpha-band coherence and increased omega complexity indicate the earliest disintegration of cortico-cortical timing, while selective delta-band hyperconnectivity—especially between premotor regions—already emerges. Stage 2 (manifest): Alpha hypocoherence deepens, and delta and high-beta synchrony extend across prefrontal, premotor, somatosensory, and occipital hubs. This low-frequency hypersynchrony is not compensatory; it correlates with impaired cognition, increased apathy, and higher motor burden. The patterns likely reflect maladaptive thalamocortical disinhibition driven by striatal GABAergic neuron loss.

#### 3.5.5. State-Dependent Abnormalities in Sleep

Electrophysiological disruption in HD is not confined to wakefulness; it extends across the entire sleep cycle and tracks both genetic load and regional brain atrophy.

Polysomnography in 38 pre-manifest carriers revealed a >15% fall in sleep efficiency (81% ± 8% vs. 96% ± 4% in controls), driven by a 40% rise in nocturnal awakenings and a doubling of the arousal index [36]. REM sleep proved particularly vulnerable: REM percentage declined from 22% to 16%, REM bout duration was shortened by 44%, and the cumulative REM deficit correlated with disease burden score (DBS) as well as early motor and affective symptoms (UHDRS-TMS ρ = −0.38; HADS-A ρ = −0.34). In symptomatic cohorts, slow-wave sleep (SWS) was consistently reduced. A CT–PSG study of 12 inpatients recorded a 33% reduction in stage N3 sleep (34 ± 11 min vs. 51 ± 14 min), alongside increased sleep-onset latency and >25% more wake time after sleep onset. These deficits were not affected by medication status [56].

Fast Fourier analyses showed that the narrow-band alpha–theta loss seen during wakefulness persists—and may intensify—during REM sleep. In pre-manifest carriers, theta power over parietal leads during REM declined by 18% (4–7 Hz SNR: 0.41 ± 0.06 vs. 0.50 ± 0.05), and REM theta attenuation scaled with DBS (β = −0.43) [36]. eLORETA mapping in a mixed-stage cohort revealed bilateral reductions in theta (4.5–7.5 Hz) and alpha (8–13 Hz) current density localized to the posterior cingulate and retrosplenial cortices during REM, while wake recordings showed focal delta excess in the primary motor cortex, and NREM recordings displayed an unexpected alpha surge in the same motor regions [52]. These NREM alpha “intrusions” coincided with increased delta connectivity across the sensorimotor network (see below) and may represent failed cortical downscaling.

Ensorimotor-network analyses using lagged phase synchronization uncovered a staged pattern of changes. Regarding the wakefulness state, pre-manifest carriers already displayed stronger delta coupling between the left and right premotor cortices (median LPS 0.44 vs. 0.29). Regarding NREM, the same premotor–somatosensory link intensified (LPS ↑ 19%), and new cross-hemispheric premotor-S1 connections emerged in the delta band; beta- and gamma-band LPS increased in proportion to motor impairment (UHDRS-TMS) and inversely with BMI [44]. Regarding REM, hypersynchrony broadened; delta LPS between premotor cortices climbed another 14%, theta LPS linked bilateral S1 cortices (LPS 0.37 vs. 0.21), and alpha LPS connected left S1 to right M1. Alpha-band REM connectivity predicted UHDMRS motor burden, CAG repeat length, and DBS [44].

Notably, neurovascular coupling deteriorated in parallel. Simultaneous EEG–fNIRS revealed that presymptomatic carriers already exhibited a 17% reduction in alpha–myogenic phase coherence (~0.1 Hz) during drowsy rest, which progressed to a 32% deficit in manifest disease [53]. Only caudate-specific, rather than global, atrophy was associated with changes in sleep EEG. The frontal-horn-to-caudate-head ratio (FH/CC) accounted for 45% of the variance in SWS duration, whereas the cerebrospinal-fluid-to-brain ratio showed no such relationship [56], highlighting a basal ganglia contribution to slow-wave generation.

Thiopentone and quinalbarbitone recordings confirmed cortical unresponsiveness; only 4 out of 16 patients produced sleep spindles, K-complexes appeared in only one, and evoked responses to sound were scarce, indicating diffuse thalamocortical disconnection even when sedation induced a sleep-like state [41].

#### 3.5.6. Task-Evoked Oscillatory Alterations

Only two studies investigated mutation carriers during active cognitive engagement, yet their convergent findings suggest that the neural architecture underlying rapid, long-range coordination deteriorates well before overt performance deficits emerge.

The first study employed a 10-item pictorial recall paradigm with a 21-electrode montage and demonstrated that although pre-manifest carriers recalled as many items as their non-carrier relatives, their cortical responses differed qualitatively: during the 4–8 s encoding window, relative alpha power fell by 21% (compared to −8% in controls), absolute alpha dropped by 0.66 μV^2^/Hz and 44% of carriers fell more than 2 SD below the control mean [45]. Theta power did not increase to compensate, implying that greater alpha ERD reflects neural inefficiency rather than a strategic reallocation to slower rhythms.

A high-density (128-channel) n-back task extended these findings across task phases with greater source precision. During both the stimulus encoding (update) and decision (readout) epochs of 2- and 3-back conditions, frontoparietal theta event-related synchronization (ERS) was nearly absent in HD gene carriers: mean ERS gain was +3% in carriers compared to +14% in controls, with the reduction spanning medial and lateral prefrontal, inferior parietal, insular, and cerebellar nodes [55]. In contrast, beta synchronization during the maintenance phase, typically associated with sustained attention, was preserved, confirming the phase-specific nature of the deficit. Although high-gamma (50–80 Hz) ERS during maintenance showed no group-level difference, its amplitude in the left cerebellum and fusiform gyrus inversely correlated with disease-burden (CAP) score among carriers, hinting at local interneuronal dysfunction that scales with genetic load. Behaviorally, carriers’ reaction times were comparable to those of controls, but their accuracy was reduced by 6–8% in the 2-back condition and 11–13% in the 3-back condition, indicating that oscillatory dysfunction precedes overt behavioral slowing.

To sum up, these tasks reveal a consistent pattern: exaggerated alpha desynchronization during basic memory encoding and blunted theta synchrony during flexible updating or retrieval. Both changes occur with minimal or subtle behavioral consequences, implicating early disintegration of cortico-striato-thalamocortical loops responsible for top-down theta coordination, while sparing local beta mechanisms that support tonic task engagement. The sensitivity of gamma-band activity to CAP score further suggests that the excitation-inhibition balance within cortico-cerebellar microcircuits is already compromised in the late pre-manifest phase, positioning task-based oscillatory metrics as promising endpoints for trials targeting cognitive preservation in HD.

#### 3.5.7. Multimodal Signatures and Machine-Learning Insights

When scalp rhythms are evaluated alongside hemodynamic signals, the electrophysiological profile gains an additional layer of insight. In the largest concurrent EEG–fMRI study to date, reduced 8–9 Hz power in 56 HTT-mutation carriers predicted a stereotyped pattern of frontostriatal and default-mode hyperconnectivity: each standard deviation decrease in low-alpha amplitude was associated with a 0.54-SD rise in Fisher-*z* correlation strength between the putamen and dorsolateral prefrontal cortex, as well as a parallel 0.41-SD increase within the posterior default-mode network. These associations remained significant after stringent false-discovery rate correction [39]. This pattern indicates that cortical slowing and fMRI hypersynchrony are mechanistically linked manifestations of thalamocortical disinhibition. A complementary EEG–fNIRS study combining electrical and optical recordings from 46 participants further demonstrated that neurovascular coupling deteriorates before clinical onset: phase coherence between parietal alpha oscillations and slow (≈0.1 Hz) oxygenation rhythms was already 17% lower in presymptomatic carriers compared to matched controls and had declined by 32% in early-manifest patients. Mixed-effects modeling confirmed a strong association with CAP score and autonomic indices, underscoring a system-wide breakdown in neurovascular unit efficiency [53].

These reproducible spectral features have proven sufficiently distinctive to support automated classification. An early artificial neural network trained on 16-channel EEGs distinguished manifest HD patients from controls with 88% accuracy and an area under the curve (AUC) of 0.92 by weighting occipital alpha suppression against frontocentral delta augmentation [37]. A more recent support vector machine, initially provided 17,290 quantitative descriptors and refined via a genetic algorithm to retain the 20 most informative features, achieved 83% accuracy (AUC 0.90). Notably, while the classifier’s raw score did not correspond to clinical severity, two post-hoc feature clusters extracted from the model correlated strongly with processing speed and motor burden, respectively, illustrating how high-dimensional spectral data can yield domain-specific biomarkers [43]. The most comprehensive classification pipeline to date integrates EEG, fNIRS, and ECG signals using an extremely randomized trees ensemble. Despite the multimodal input, EEG features—particularly parieto-temporal alpha loss recorded at P8—dominated model importance, resulting in a cross-validated accuracy of 91% with an AUC of 0.963 [57].

Taken together, these multimodal observations and machine learning results converge on a coherent mechanistic narrative: the earliest electrophysiological consequence of mutant huntingtin is a focal depletion of low-alpha generators, which disrupts synchrony between neural and vascular oscillations and induces compensatory but ultimately maladaptive large-scale network hyperconnectivity. As this signature is both stable and quantifiable, shallow learning algorithms can be used to detect using fewer than 30 spectral features and translate it into continuous indices that reflect genotype load, cognitive function, and motor impairment, offering a practical, non-invasive endpoint for preventive and therapeutic interventions in HD.

**Table 1:** EEG studies included in this review.

Study	Participants & Stage	EEG Paradigm/Analysis	Core Spectral/Network Findings	Clinical & Biomarker Take-Aways
[36]	38 Pre-HD vs. 36 ctrls	2-night PSG + REM spectral power	↑ Sleep fragmentation; ↓ REM %, REM bout, REM-theta (4–7 Hz)	REM measures track Disease-Burden-Score; no metabolic change
[37]	13 manifest, 7 at-risk, 13 ctrls	Awake eyes-closed; 16-ch FFT → ANN	↓ Alpha, ↑ δ/θ; ANN best (92% overall)	ANN output continuous with yrs-to-onset (r ≈ −0.7)
[38]	10 HD, 25 DAT, 20 ctrls	16-ch FFT; %EEG Power = α/(α + θ)	HD & DAT both show ↓ %Power	70% sensitivity, 90% specificity for HD
[39]	45 Pre-HD, 11 EMHD, 41 ctrls	Resting EEG + rs-fMRI; 1-Hz bins	↓ 7–9 Hz & broad α; ↑ β2, δ	8–9 Hz power ↘ with CAG, cognition; EEG-α links to hyper-rsFC
[40]	55 manifest (stg 1–4) vs. 55 ctrls	V-EEG + LORETA	Global ↓ θ/α/β; ↑ δ in right OFC (adv stg)	α drop is trait marker; θ/α power ↘ with MMSE, ↑ motor
[41]	16 manifest	Rest & barbiturate-sleep EEG	Low-voltage (<10 μV); sparse spindles	“Flat” EEG precedes death; sleep features most sensitive
[42]	95 manifest (1950-70 data)	Routine clinical EEG	33% “low-voltage” (<10 μV)	Low-voltage linked to dementia + FHx; persists over years
[43]	6 Pre-, 20 early, 25 ctrls	qEEG (19-ch, 17 k features) → SVM	Global power ↓; α splitting; 22 Hz resonance	SVM 83% acc.; indices map to SDMT & UHDRS
[44]	23 manifest vs. 23 ctrls	eLORETA lagged phase-sync across sleep	↑ δ connectivity wake + NREM; widespread low-freq hyper-sync REM	Connectivity strength ∝ motor score, CAG
[45]	16 Pre-HD vs. 13 non-carriers	Working-memory EEG	Rest = n.s.; WM: ↓ α power	44% carriers fall > 2 SD; no perf. drop yet
[46]	55 manifest vs. 55 ctrls	3-min V-EEG mapping	↑ δ/θ, ↓ α/β, slowed centroids	Power shifts tie to MMSE & motor; meds minor effect
[47]	27 HD (3 pre) vs. 15 ctrls	35-ch QEEG; AP gradients	Loss of posterior-α & anterior-δ gradients	Gradient loss tracks TFC, TMS, CAG; seen pre-HD
[48]	20 unmed mild-mod vs. 20 ctrls	eLORETA + microstates	↓ global lagged-FC; ↑ μ-state A/B, ↓ C/D	μ-state A ↑ with UHDRS; Ω-complexity ↑ with stage
[49]	18 Pre-, 49 manifest, 38 ctrls	61-ch eLORETA LPS	Manifest: widespread hypersynchrony (δ + α + β)	Hyper-LPS ↘ SDMT & Stroop; pre-HD subtle
[50]	29 Pre-HD vs. 29 ctrls	1-Hz sub-band FFT	↓ 7–8 & 8–9 Hz; delta/theta ↑	7–8 Hz–4–5 Hz diff r = −0.60 to CAG; ties to fluency
[51]	16 mild-mod vs. 8 ctrls	8-lead PSA	↓ α, ↑ δ/β; frontal θ peak slows	Spectra map to QNE, Stroop, WCST; beta ↑ → exec deficit
[52]	23 manifest vs. 23 ctrls	eLORETA: wake & sleep	Wake: ↑ δ motor ctx; NREM: ↑ α motor, ↓ θ limbic; REM: limbic α/θ ↓	Motor-cortex power ∝ UHDRS; state-specific patterns
[53]	13 pre, 15 symp vs. 62 ctrls	Simult. EEG + fNIRS	α power ↓; EEG-fNIRS coherence ↓ even pre-HD	NVU dysfunction appears presymptomatically
[54]	11 pre/11 early vs. 20 ctrls	Periodic-only EEG; wPLI	-	θ power r = 0.66 with SDMT; no apathy link
[55]	7 pre, 9 early vs. 16 ctrls	hdEEG during 2/3-back	-	Task-θ deficit precedes big behavioral loss
[56]	12 manifest	Nocturnal sleep-EEG + CT	-	Links basal ganglia loss to sleep quality
[57]	27 HD vs. 36 ctrls	20 min EEG/ECG/fNIRS; 948 feat; ERT	EEG (P8) dominates; 91% accuracy	Low-cost multimodal classifier; EEG most informative

Abbreviations: α/β/γ/δ/θ—Alpha/Beta/Gamma/Delta/Theta EEG bands, acc.—Accuracy, ANN—Artificial Neural Network, AP gradient—Anterior–Posterior gradient, CAG—Cytosine–Adenine–Guanine repeat length in HTT, Crtls/ctrls—Control subjects, CT—Computed Tomography, DAT—Dementia of the Alzheimer’s Type, eLORETA/LORETA—(Exact) Low-Resolution Electromagnetic Tomography, EMHD—Early-Manifest Huntington’s Disease, ERT—Extremely Randomized Trees classifier, FFT—Fast Fourier Transform, FHx—Family History, fNIRS—Functional Near-Infrared Spectroscopy, fMRI/rs-fMRI—(Resting-State) Functional Magnetic Resonance Imaging, HD—Huntington’s Disease, hdEEG—High-Density EEG, LORETA—see eLORETA, LPS—Lagged Phase Synchronization, MMSE—Mini-Mental State Examination, n.s.—Not Significant, NVU—Neurovascular Unit, OFC—Orbitofrontal Cortex, Ω-complexity—Omega Complexity (spatial EEG complexity), PSA—Power Spectral Analysis, PSG—Polysomnography, Pre-HD—Premanifest Huntington’s Disease, QEEG—Quantitative EEG, QNE—Quantitative Neurological Examination, REM—Rapid Eye Movement sleep, SVM—Support Vector Machine, SD—Standard Deviation, SDMT—Symbol Digit Modalities Test, SWS—Slow-Wave Sleep, TFC—Total Functional Capacity (UHDRS subscale), TMS—Total Motor Score (UHDRS), UHDRS—Unified Huntington’s Disease Rating Scale, V-EEG—Vigilance-Controlled EEG, wPLI—Weighted Phase-Lag Index, WM—Working Memory, yrs—Years.

## 4. Discussion

Huntington’s disease is a severe, incurable neurodegenerative disorder with a complex pathophysiology. Neuroimaging methods are essential to elucidate its impact on the brain. This mechanistic review examines the application of EEG in HD. Twenty-two studies were included in the analysis. These studies vary considerably in methodology, patient cohorts, signal analysis techniques, and other parameters. Nevertheless, the incorporation of multimodal neuroimaging approaches indicates that EEG research in HD is an intensively developing field. The compiled studies reveal several consistent patterns and findings, demonstrating that EEG can serve as a valuable tool for diagnosing HD and monitoring functional decline.

### 4.1. Alpha Oscillations—A New Neurophysiological Biomarker in HD

EEG studies converge on a remarkably consistent observation: loss of cortical alpha-band activity—especially within the narrow 7–9 Hz range—is one of the earliest and most progressive electrophysiological signatures of HD. In two independent resting-state cohorts of gene-positive but still asymptomatic carriers, power in the 7–8 Hz and 8–9 Hz frequency bins was already reduced compared to controls, with medium-to-large effect sizes and negative correlations with CAG repeat length and disease-burden score [39,50]. With the onset of early-manifest disease, this focal reduction becomes more widespread: global alpha power decreases by approximately 30–50%, and the normal posterior dominant rhythm becomes indistinct [38,40,46,51]. Power-spectral ratios combining alpha-band attenuation with increases in theta and delta power reach sensitivities of 68–83% and specificities of 83–90% for distinguishing patients from controls [37,38,43]. In an artificial neural network classifier, alpha suppression carried the greatest discriminative weight; when trained on manifest cases, the model assigned intermediate “probability-of-disease” values to pre-manifest carriers, which scaled inversely with estimated years to onset [37]. A larger multimodal machine-learning pipeline integrating EEG, ECG, and fNIRS extracted 948 peri-epoch features and achieved 91% accuracy with an extremely randomized trees model; once again, the most informative features were derived from alpha-band EEG channels, particularly P8 [57].

Physiological plausibility is supported by source-space and multimodal studies. Exact LORETA mapping reveals that alpha attenuation originates across widespread cortical grey matter and intensifies over frontostriatal projections. In contrast, delta increases localize to the orbitofrontal cortex only in advanced stages, implying a staged breakdown of cortico-striato-thalamocortical circuits [40]. Simultaneous EEG–fMRI recordings demonstrate that lower alpha power covaries with hyperconnectivity in default-mode, frontal-executive, and putaminal networks, a pattern interpreted as thalamocortical disinhibition and compensatory up-regulation [39]. Complementary connectivity metrics, based on lagged phase synchronization and the weighted phase-lag index, show that manifest HD is characterized by widespread delta hypersynchronization and reduced alpha coherence; the latter is correlated with slower processing speed (SDMT) and higher apathy scores [49,54].

Behaviorally, alpha indices track both cognitive and motor function. Among pre-manifest carriers, stronger 8–9 Hz power predicts better verbal fluency [39,50]. In symptomatic individuals, reduced alpha power and a slower alpha centroid are associated with higher UHDRS motor scores, lower Mini-Mental State Examination scores, and reduced performance in activities of daily living [40,46,51]. During cognitive challenges, carriers display an exaggerated decrease in alpha power (event-related desynchronization) despite preserved task accuracy, suggesting that inefficient neural recruitment precedes overt performance decline [45]. This over desynchronization is similarly evident during the update and readout phases of an n-back task in early-manifest HD [55].

These effects are not attributable to medication. Drug-free sub-group analyses replicate the alpha reductions [40,46], and the only consistent pharmacological effect—slight slowing of the dominant alpha peak—accounts for less than 5% of the variance [46]. Nor are these findings indicative of generic dementia: in Alzheimer’s disease, the dominant alpha frequency typically shifts downward in early stages, whereas in HD, the peak remains stable until late in the disease course, with early deficits appearing as a selective erosion of the 7–9 Hz flank [38]. At the other extreme of the spectrum, a “low-voltage” EEG phenotype (<10 μV across bands) that may develop after a decade of illness can obscure all oscillations, thereby saturating alpha metrics and reducing their dynamic range [42].

Taken together, the data meet classical biomarker criteria. (i) Early detectability: alpha abnormalities appear at least a decade prior to clinical diagnosis [39,50]. (ii) Biological gradient: alpha attenuation scales with CAG repeat length and disease burden score [39,50]. (iii) Clinical relevance: Alpha metrics correspond to cognitive decline, executive dysfunction, and motor deterioration [40,46,51,54]. (iv) Technical feasibility: resting-state EEG with automated spectral analysis is inexpensive, supports multicenter acquisition, and directly feeds into machine-learning classifiers with over 80% accuracy [37,43,57]. (v) Specific pathophysiology: source-localization and network analyses link the signal to thalamocortical disinhibition and striatal degeneration [39,40,49]. Therefore, quantitative alpha-band metrics—particularly power in the 7–9 Hz window and the preservation of normal posterior gradients—emerge as sensitive, functionally meaningful, and scalable biomarkers for monitoring preclinical progression and evaluating therapeutic response in HD. The potential mechanisms underlying alpha power reduction in HD are explored in more detail in Section 5 of this paper.

### 4.2. Other Oscillatory Bands: Delta, Theta, Beta, and Gamma

A progressive increase in cortical delta power is a well-established hallmark of symptomatic HD. Broad-band mapping has revealed widespread delta augmentation—most notably in frontotemporal and centro-parietal regions—accompanied by slowed frequency centroids and reduced total power in 55 patients [46]. Low-resolution tomography localized the earliest delta rise to the right orbitofrontal cortex and subsequently tracked its expansion across frontal and limbic areas as patients advanced from Shoulson stage 1 to stages 3/4 [40]. Source-space EEG confirmed a focal delta hotspot in primary motor areas during quiet wakefulness, with magnitude scaling positively with disease duration [52]. Machine-learning studies have also identified significantly elevated delta activity at prefrontal poles (Fp1/Fp2) and along sections of the sensory–motor strip, contributing to an overall classification accuracy of 83% [43]. Importantly, delta power (and the associated loss of the delta–alpha anterior–posterior gradient) tracks clinical severity: higher delta correlates with lower Total Functional Capacity, increased UHDRS-TMS, and greater CAG-adjusted age, in both early-manifest and pre-manifest cohorts [39,47].

Theta-band findings are more variable and state-dependent. In conventional FFT-based studies, theta power typically increases in parallel with delta, particularly over frontal electrodes in manifest HD, and contributes additional discriminative value in neural-network classifiers [37,46]. Elevated theta also correlates positively with CAG repeat length [39]. In contrast, studies that separate periodic from aperiodic activity report a reduction in pure oscillatory theta power in late pre-manifest or very early HD, most prominently during eyes-closed rest across frontal, temporal, and occipital sites [54]. Power-spectral analyses with high spatial resolution similarly observed reduced theta power and a slower peak theta frequency at frontal electrodes F3/F4 in mildly affected young patients; these reductions predicted poorer Stroop test performance and greater motor impairment [51]. These findings suggest that theta power may initially decline due to failed compensatory mechanisms at the alpha/theta boundary, before rising again as neuronal deafferentation progresses.

Beta abnormalities are heterogeneous and frequency-specific. Most resting-state studies report a global decline in low beta power (β1; ~13–20 Hz), occurring alongside the alpha reduction [38,40,46]. However, two converging findings indicate a selective increase in high-beta activity (β2; 20–30 Hz): (i) early-manifest HD patients showed significantly higher β2 power than both pre-manifest carriers and controls [39]; (ii) quantitative PSA in unmedicated patients detected excess beta power and faster beta peak frequency over the left temporal cortex, correlating with executive dysfunction, including impaired performance on Trial-Making and WCST tasks (perseverative errors) [51]. Connectivity analyses support this distinction: exact LORETA and weighted-PLI analyses reveal hypersynchronization in beta-2/beta-3 bands involving prefrontal, premotor, and somatosensory areas, with over-coupling linked to slower Stroop and SDMT performance or higher disease burden [49,54]. Collectively, the data suggest a “U-shaped” trajectory in beta oscillations—early compensatory increases in fast beta superimposed on a gradual decline of lower beta generators as corticostriatal loops degenerate.

Results in the gamma band are scarce but convergent on subtle, task-dependent changes. Resting-state eLORETA analyses found no group-level differences in gamma power [52], and gamma-band coherence between HD patients and controls is largely preserved, except for a mild reduction in long-range phase-locking in manifest cases [53,54]. Under cognitive load, however, high-gamma ERS (50–80 Hz) during working-memory maintenance declines proportionally to the CAP disease burden score, particularly within the left cerebellum and fusiform gyrus [55]. A unique spectral feature at 22 Hz (straddling the low-gamma and fast-beta boundary) has been identified in the right temporal cortex of HD carriers but is absent in controls, potentially serving as an early-stage spectral hallmark [43]. Since gamma activity is closely linked to the function of inhibitory interneurons—which are especially vulnerable in HD—these task-induced reductions likely reflect local circuit dysfunction rather than global network slowing.

### 4.3. Outcomes from Multimodal Neuroimaging Methods

Multimodal studies combining EEG with other neuroimaging techniques are particularly informative, enabling the translation of the brain’s electrical activity into complementary functional parameters. In one such study [39], in addition to EEG findings of reduced power in the 7–9 Hz band, HTT mutation carriers exhibited reduced positive connectivity within frontal networks, including the superior frontal gyrus (SFG), lateral prefrontal cortex (LPFC), and anterior cingulate cortex (ACC), as measured by functional magnetic resonance imaging (fMRI). Reduced connectivity was also observed between frontal and cerebellar regions. Furthermore, negative resting-state functional connectivity (rsFC), typically seen between the hippocampus and occipital regions and between the cerebellum and posterior cortex, was diminished in mutation carriers, indicating a widespread disruption of both excitatory and inhibitory network dynamics. Importantly, regression analyses integrating EEG and fMRI data demonstrated that reduced alpha power was significantly associated with increased rsFC within several large-scale brain networks, particularly those involving putamen-frontal circuits, the default mode network (DMN)**,** and hippocampal-temporal connections. These findings suggest that EEG slowing—interpreted as a marker of cortical disinhibition and impaired thalamocortical regulation—is mirrored by hyperconnectivity in fMRI, potentially reflecting early compensatory neural responses. Taken together, this study demonstrates that alterations in EEG spectral power, especially in the 7–9 Hz frequency band, along with disrupted fMRI connectivity patterns, are detectable even in the preclinical stages of HD and become more pronounced with disease progression.

Another important application of multimodal EEG is its integration with fNIRS neuroimaging. Study [57] was the first to aim at developing a low-cost, physiologically grounded, and accurate diagnostic system for HD using non-invasive biomedical signals—specifically EEG, ECG, and fNIRS—combined with extensive time- and frequency-domain feature extraction and shallow machine learning models. Although this study did not measure brain activity outcomes directly, it demonstrated that combining EEG with fNIRS is technically feasible and applicable in research on HD patients.

A subsequent study [53] aimed to determine whether the efficiency of the neurovascular unit (NVU)—the coordinated system linking neuronal activity to cerebral blood flow—is compromised in HD, including its presymptomatic stage. The authors hypothesized that altered neuronal and vascular oscillatory dynamics, as well as reduced neurovascular coupling, could serve as functional biomarkers of HD progression. To test this, they simultaneously recorded electrical and vascular brain signals using EEG and fNIRS, alongside heart rate (ECG) and respiratory measurements, in both symptomatic and presymptomatic HD gene carriers, as well as age-matched healthy controls.

Cardiovascular data showed that symptomatic patients exhibited significantly elevated heart and respiration rates compared to controls. However, coherence between intrinsic heart rate (IHR) and brain oxygenation (from fNIRS) was significantly reduced in both the presymptomatic and symptomatic groups, particularly in the myogenic and respiratory frequency bands. Notably, the presymptomatic group already displayed reduced coherence across multiple probe pairs, suggesting an early disruption of cardiorespiratory coupling.

Brain oxygenation analysis via fNIRS further revealed that the presymptomatic group had a significantly reduced power in neurogenic and myogenic frequency bands across several cortical regions, indicating impaired vasoreactivity. By contrast, the symptomatic group displayed elevated respiratory-band power, possibly reflecting compensatory vascular remodeling. Despite these alterations, spatial coherence between oxygenation signals (i.e., synchronization of vascular rhythms across cortical regions) was reduced in the symptomatic group, showing that structural vascular changes did not restore functional efficiency.

Neurovascular coupling, measured as phase coherence between EEG and fNIRS signals, was reduced in both HD groups, particularly in the myogenic band (~0.1 Hz), which reflects vascular smooth muscle response. These reductions were especially pronounced in occipital and parietal regions, areas previously known to undergo early atrophy in HD. In the group with severe chorea, neurovascular coherence was further reduced, suggesting progressive functional disconnection with disease advancement. The authors interpreted the diminished neurovascular phase coherence as evidence of NVU dysfunction in HD, likely driven by a combination of vascular remodeling, impaired metabolic signaling, and astrocyte-mediated disruptions in neurovascular communication. Importantly, these alterations were detectable during the presymptomatic phase, even in the absence of major changes in EEG power, suggesting that vascular dysfunction may precede overt neuronal degeneration.

A third form of multimodal neuroimaging combined EEG with computed tomography (CT). In study [56], all participants underwent cranial CT scans, which were analyzed using standardized anatomical markers and density thresholds. These included the frontal horn-to-caudate head ratio (FH/CC), used to assess local caudate atrophy, and cerebrospinal fluid-to-brain ratio (CSFBR), an index of global brain atrophy. CT imaging confirmed that most patients exhibited both caudate-specific and generalized cerebral atrophy, consistent with established HD pathology. Quantitatively, the FH/CC ratio was significantly elevated, reflecting reduced caudate volume, while CSFBR values confirmed diffuse brain volume loss.

Importantly, both atrophy measures correlated significantly with disease duration, but not with age, suggesting that the observed morphological changes were disease-specific rather than age-related. Global cerebral atrophy, as measured by CSFBR, was significantly associated with clinical metrics, including global clinical assessments and psychopathological symptoms, particularly anergia and thought disturbances on the Brief Psychiatric Rating Scale (BPRS). In contrast, caudate atrophy (FH/CC) did not correlate with these psychopathological variables. No significant relationships were found between atrophy measures and motor symptoms, as measured by the Tardive Dyskinesia Rating Scale (TDRS), nor between any CT measures and BPRS anxiety/depression scores.

The authors interpreted these findings as supporting a functional role for the basal ganglia—specifically the caudate nuclei—in sleep regulation, possibly via connections to thalamocortical and arousal networks. This interpretation is further supported by prior PET studies showing altered caudate metabolism during sleep and in conditions with overlapping symptomatology (e.g., depression). Notably, while cortical atrophy generally reduces EEG amplitude and could artifactually influence SWS measurements, this study found that SWS reduction correlated specifically with caudate atrophy, and not with global brain volume loss. This suggests that the effect was physiologically meaningful rather than artifactual. The implications of these sleep-related findings are elaborated in the subsequent section.

Taken together, the three multimodal datasets converge on a coherent pathophysiological cascade that begins in the NVU, progresses through cortico-striato-thalamocortical circuits, and ultimately leads to macroscopic atrophy and sleep disruption, as captured by CT and polysomnography.

Mutant huntingtin is expressed not only in neurons but in endothelial cells, pericytes, and astrocytes—key cellular components responsible for coupling cerebral blood flow to metabolic demand. Human fNIRS-EEG recordings show that even presymptomatic carriers have attenuated 0.1 Hz myogenic oscillations and markedly reduced phase coherence between oxyhemoglobin signals and both heart-rate variability and EEG rhythms. These findings indicate early impairment in vascular smooth-muscle responsiveness and neurovascular coupling, preceding detectable neuronal loss. In vivo MRI studies corroborate widespread small-vessel remodeling, impaired CO_2_ cerebrovascular reactivity, and perivascular-space enlargement, all linked to mHTT-driven WNT signaling disruptions and astrocytic dysfunction [58,59].

Cortical disinhibition manifests in scalp EEG as a selective drop in low-alpha (7–9 Hz) power, accompanied by increases in slower theta and delta components. Given that alpha rhythms normally gate information flow, their reduction permits greater but less specific long-range synchronization. Consequently, resting-state fMRI reveals hyperconnectivity within putamen–frontal, default-mode, and hippocampal–temporal networks, with connectivity strength inversely related to alpha power. This pattern is best interpreted as a compensatory—but metabolically costly—recruitment of parallel neural pathways to maintain cognitive and functional capacity amid declining local processing efficiency.

### 4.4. Microstate Outcomes

Study [48] investigated resting-state electroencephalographic alterations in patients with HD by assessing both global brain dynamics and EEG microstate patterns, with the aim of identifying electrophysiological markers of disease-related neural dysfunction. The researchers focused on two principal domains: (1) global EEG characteristics—specifically lagged functional connectivity and omega complexity—as measures of inter-regional synchrony and signal complexity, respectively, and (2) microstate properties, reflecting the temporal dynamics of transient, topographically stable brain states believed to underlie core functional processes.

The analysis yielded several key findings. First, global lagged connectivity was significantly reduced in HD patients compared to controls (mean connectivity HD = 0.42 vs. control = 0.70), indicating substantial disruption in the synchronization of brain activity across regions. In contrast, omega complexity did not differ significantly between groups; however, within the HD group, it correlated positively with disease severity (Shoulson stage), suggesting that patients with more advanced disease displayed more spatially fragmented, independent cortical activity.

Second, microstate analysis showed a marked reorganization of temporal EEG patterns in HD. Specifically, microstate class A showed significantly increased coverage and occurrence in HD patients, indicating that this class dominated more of the resting EEG and occurred more frequently. Microstate class B showed a similar increase in both coverage and occurrence. In contrast, microstate class C was significantly reduced in occurrence and showed a trend toward reduced coverage. Microstate class D demonstrated decreased coverage and occurrence, indicating a diminished presence of this brain state in HD. Correlation analyses showed that higher class A coverage and occurrence were positively associated with disease stage and UHDRS-TMS, suggesting a link between sensorimotor network overrepresentation and motor dysfunction. Conversely, reduced class C coverage and duration correlated with lower MMSE scores and worse cognitive performance on Stroop and SDMT tests. Higher omega complexity was also associated with poorer cognitive outcomes (lower MMSE, Stroop, and verbal fluency (VF) scores), reinforcing the interpretation that disease progression entails increasing cortical disintegration. While lagged connectivity was globally reduced in HD, it did not strongly correlate with specific clinical measures, suggesting it reflects generalized network dysfunction rather than symptom-specific impairment.

These alterations were interpreted as reflecting a breakdown in coordinated brain activity in HD. The decrease in global functional connectivity and increase in omega complexity point to a shift from coherent, integrated brain states toward fragmented, functionally disconnected networks. Changes in microstate dynamics—particularly the increase in classes A and B and reduction in classes C and D—indicate an altered temporal structuring of neural processing, consistent with both pathological degeneration and compensatory reorganization.

Resting-state EEG in HD thus reveals a coherent pathophysiological sequence that begins with structural disconnection, progresses to desynchronized neural dynamics, and culminates in a characteristic redistribution of canonical microstate classes. Diffusion tensor imaging demonstrates early and progressive loss of callosal and long-range cortico-cortical fibers, indexed by reduced fractional anisotropy and increased radial diffusivity; meta-analytic data show that the magnitude of these abnormalities scales with CAG repeat length and motor impairment, establishing the anatomical basis for large-scale functional decoupling [60,61]. Functional MRI studies corroborate this interpretation, documenting widespread hypoconnectivity between associative cortical hubs in manifest HD, despite occasional relative hyperconnectivity within residual sensorimotor loops [62,63]. An EEG study reported a ~40% reduction in lagged phase-synchrony—a metric unaffected by volume conduction and therefore a direct electrophysiological readout of impaired long-range integration. As structural and functional links are lost, cortical columns become more autonomous, a transition captured by omega complexity, which quantifies the spatial dimensionality of scalp EEG via the Shannon entropy of the covariance matrix eigen-spectrum. Methodological analyses confirm that omega increases as synchrony declines [64,65]. In the HD cohort, group-level omega did not yet differ from controls, but it increased monotonically with Shoulson stage, mirroring findings in schizophrenia [66].

Microstate segmentation provides additional temporal resolution. Simultaneous EEG–fMRI studies have linked microstate class A to sensorimotor and inner speech networks, class B to early visual regions, class C to salience and interoceptive networks, and class D to the frontoparietal control system [67,68,69,70]. In HD, an increased coverage and occurrence of classes A and B, alongside reduced presence of classes C and D, reflect a shift toward sensorimotor dominance and executive disengagement. These microstate alterations selectively correlate with clinical status: elevated class A activity aligns with more advanced motor symptoms and disease stage, whereas diminished class C activity correlates with impaired cognition (MMSE, Stroop, and SDMT scores). A noisy-network attractor model suggests that the weakening of hub nodes flattens the global neural state space while deepening attractor basins associated with structurally preserved networks. The degeneration of striatal indirect-pathway outputs and callosal fibers may therefore stabilize sensorimotor microstates (A/B) and erode salience and executive microstates (C/D) [71]. This temporal bias towards sensorimotor states provides a mechanistic explanation for the clinical combination of hyperkinetic movement disorders and executive slowing observed in HD.

Moreover, the dissociation between static connectivity, which saturates once hub integrity is sufficiently compromised, and dynamic microstate metrics, which remain sensitive to residual network-specific capacity, accounts for their differing relationships with symptom severity. The HD microstate profile (decreased connectivity, increased omega, elevated A/B, reduced C/D) also distinguishes it from other neurodegenerative and psychiatric conditions: for example, Alzheimer’s disease shows reduced omega and increased class C, while schizophrenia features elevated omega with shortened microstate durations across all classes [66,72,73]. Finally, the normalization of functional coupling by sigma-1-receptor modulation with pridopidine underscores the translational potential of EEG microstate and complexity parameters as biomarkers for disease progression and therapeutic response [62]. In summary, converging evidence supports a mechanistic chain in which mutant-huntingtin–induced white matter degeneration disrupts corticostriatal and interhemispheric pathways, reduces large-scale synchrony, increases cortical spatial complexity, and reshapes the attractor landscape such that sensorimotor microstates predominate and control-related microstates recede. This electrophysiological trajectory provides a crucial link between molecular pathology and clinical manifestation in HD.

### 4.5. Sleep Outcomes

Several EEG studies have examined electrical activity during sleep in HD patients, revealing early and progressive alterations in sleep architecture and physiology.

In study [36], significant sleep abnormalities were observed even in the pre-manifest group. These included increased sleep fragmentation, elevated arousal indices, reduced REM sleep percentage, shortened REM bout duration, and diminished theta power (4–7 Hz) during REM sleep. These deficits correlated significantly with the DBS, a composite measure incorporating age and CAG repeat length. Additionally, objectively measured sleep abnormalities were associated with early changes in cognition, affect, and motor function, suggesting that sleep disruption may emerge alongside other subtle non-motor features prior to formal disease onset. In contrast, no metabolic abnormalities were detected in the pre-manifest group. Measures such as basal metabolic rate, total energy expenditure (both in-lab and under free-living conditions), activity-related energy expenditure, and hormonal levels (including cortisol, testosterone, leptin, and vitamin D) did not differ significantly between pre-manifest carriers and controls. These findings suggest that although weight loss is a recognized early feature of manifest HD, it is not driven by detectable metabolic dysfunction at the pre-manifest stage.

In study [41], in addition to standard resting EEG recordings, the researchers conducted barbiturate-induced sleep assessments. Thiopentone-induced EEGs showed partial progression through expected sleep-related rhythms. Thirteen of sixteen patients exhibited some degree of “sleep progression,” moving from beta and theta to delta frequencies. However, in four of these cases, delta activity failed to fully emerge. Where delta was present, it was typically low in amplitude and irregular, not matching the depth of sleep inferred from clinical observation. EEG amplitude increased during infusion in some patients—exceeding 10 μV in six patients and reaching up to 40 μV in one—but returned to baseline during recovery. Despite these changes, sleep-specific EEG features were sparse: spindles were observed in only four recordings, K-complexes in one, and evoked responses to sound in only two patients, both of which appeared as isolated sharp waves. EEGs recorded after quinalbarbitone administration resembled those induced by thiopentone, although in four patients, no fast (beta) activity was detected. Only one of these had also failed to show sleep progression under thiopentone, suggesting inter-individual variability in cortical responsiveness to different barbiturates. Again, spindles and K-complexes were rare and inconsistently distributed among participants.

Study [52] investigated alterations in EEG spectral power across non-REM (NREM) and REM sleep using eLORETA to localize cortical activity. During REM sleep, HD patients demonstrated bilateral reductions in both theta and alpha power in limbic and posterior association areas, specifically BAs 23, 29, 30, and 31. Notably, no changes were detected in the motor cortex during REM, consistent with the absence of increased motor activity in HD during this sleep stage. These limbic deficits may underlie the memory and affective disturbances commonly seen in HD, as slow oscillations during REM are implicated in memory consolidation and emotional processing. Correlation analyses revealed that EEG power in BA 4 (primary motor cortex) was positively associated with age and disease duration during wakefulness, and with UHDMRS scores during NREM sleep. In REM sleep, BMI and disease duration were also correlated with motor cortex spectral activity. No significant correlations were found between EEG power and CAG repeat length, suggesting that EEG spectral changes are more closely aligned with phenotypic expression than with genetic burden. The authors propose that these state-dependent EEG abnormalities—particularly the increased delta during wakefulness and elevated alpha during NREM—may reflect either motor cortex dysfunction or compensatory mechanisms attempting to modulate abnormal motor output. The absence of REM-related motor cortex changes along with the bilateral (but right-dominant) distribution of abnormalities supports the hypothesis of altered interhemispheric motor regulation in HD. Moreover, the reductions in limbic theta and alpha activity during REM and NREM sleep may contribute to sleep-related cognitive impairments, including memory loss, given the established role of these oscillations in neural plasticity. Although most patients were receiving medication (e.g., neuroleptics, antiepileptic mood stabilizers, and benzodiazepines), the focal nature of EEG changes, as opposed to diffuse alterations, suggests that these findings more likely reflect underlying disease pathology rather than pharmacological effects.

Study [56] investigated the relationship between brain structural changes—measured via CT—and sleep EEG parameters in patients with HD, aiming to clarify how local and global atrophy relate to specific alterations in sleep architecture. The researchers focused particularly on atrophy of the caudate nuclei and broader global cerebral atrophy and assessed their associations with both nocturnal sleep structure and clinical psychopathology. Sleep EEG recordings were conducted over a standard 8-h night following an adaptation period. The results revealed that HD patients experienced reduced sleep efficiency, primarily due to increased nocturnal wakefulness and a greater number of awakenings. They also showed longer sleep onset latency and a trend toward decreased SWS, although the amount of REM sleep and its structure (latency and density) did not differ significantly from controls. Drug treatment did not significantly influence sleep outcomes, as both medicated and unmedicated patients displayed similar abnormalities. Critically, correlation analyses linked specific brain morphological changes to sleep EEG disruptions. A lower FH/CC ratio, indicating more severe caudate atrophy, was strongly correlated with reduced SWS and increased time spent awake, whereas CSFBR, the measure of global atrophy, showed no significant relationship to sleep parameters. Thus, this study provided evidence that localized degeneration of the caudate nuclei is more tightly associated with sleep disturbances in HD than generalized brain volume loss.

The degeneration of the caudate-putamen—the primary lesion in HD—removes the GABAergic “brake” that medium spiny neurons normally impose on the indirect basal ganglia pathway. When this striatal output fails, the globus pallidus externus and thalamic reticular nucleus (TRN) become relatively overactive; thalamic relay cells therefore linger in a tonic, wake-like firing mode instead of transitioning into the burst pattern that opens the cortical “sleep gate”. The immediate electrophysiological consequence is the polysomnographic picture seen even in pre-manifest carriers: prolonged sleep-onset latency, frequent awakenings, and loss of slow-wave activity, all of which scale with computed tomographic measures of caudate atrophy [74].

Because spindles and K-complexes originate from reciprocal bursting between the TRN and thalamocortical neurons [75], the same circuit imbalance explains why barbiturate-induced recordings in HD show only rudimentary beta-to-theta progression and a near-absence of spindles: pharmacological GABA_A potentiation cannot restore an intrinsic pacemaker that has lost its striatal modulation, so delta oscillations appear small, irregular, or fail to emerge at all. Recent work in rodents confirms that coherent spindle trains require intact TRN–relay reciprocity [76].

Concurrently, HD pathology spreads to the lateral hypothalamus, where orexin (hypocretin) neurons that normally stabilize the VLPO/arousal flip-flop [77,78] are lost; a CSF study showed reductions of 72% in mice [79]. Without orexinergic tone, the boundary between vigilance states becomes labile, producing the fragmented REM bouts and high arousal indices reported in gene carriers long before overt motor signs appear.

Further upstream, mutant huntingtin impairs hippocampal [80], retrosplenial [81], and posterior cingulate circuits [82] that generate the theta rhythm of REM sleep [83,84,85]. Exact low-resolution tomography, therefore, reveals bilateral θ/α power loss in Brodmann areas 23 and 29–31, while the motor cortex—functionally silenced during REM [86]—remains unaffected. The same retrosplenial regions in healthy brains fire phase-locked to hippocampal theta and are critical for memory consolidation; disruption would be expected to impair overnight mnemonic processing, consistent with early cognitive complaints in HD [87].

Taken together, the EEG abnormalities across wake, NREM and REM can be traced to a single, vertically organized circuit failure: striatal neuron loss destabilizes the basal ganglia–thalamic “gate,” thalamic dysrhythmia abolishes spindles and slow waves, orexin depletion removes the chemical lock on state transitions, and limbic circuitry degeneration strips REM of the theta/alpha oscillations required for affective and declarative memory. Each layer of this cascade reproduces one facet of the heterogeneous sleep phenotype in HD while leaving whole-body metabolic rate untouched, explaining why weight loss emerges only later as hyperkinesia and neuroendocrine changes intensify.

## 5. Alpha Oscillations and Excitation and Inhibition (E/I) Imbalance in Huntington’s Disease

Huntington’s disease is characterized by a progressive breakdown of cortico–striato–thalamo–cortical (CSTC) networks [88]. In healthy CSTC circuits, cortical pyramidal neurons send glutamatergic (excitatory) inputs to striatal medium spiny neurons (MSNs), while local inhibitory interneurons—notably parvalbumin-expressing fast-spiking interneurons—provide feed-forward GABAergic control of MSNs [89,90]. MSNs relay output via the basal ganglia (direct and indirect pathways) back to the thalamus and cortex [91], closing the loop. Inhibition within this loop arises from multiple sources: feed-forward inhibition by striatal interneurons receiving cortical excitation [92] and recurrent collaterals of MSNs themselves [93]. Key regulatory mechanisms include cortical inputs, where Layer V pyramidal neurons provide glutamatergic drive to both MSNs and striatal interneurons [94,95], and feed-forward inhibition, where striatal GABAergic interneurons (especially PV+ fast-spiking cells) receive cortical excitation and inhibit MSNs, shaping their timing [90]. Together, these mechanisms maintain a balanced excitatory/inhibitory (E/I) state. The disruption of this balance can lead to hyperexcitable networks or excessive inhibition, with profound effects on motor, cognitive, and psychiatric function.

In HD, multiple lines of evidence indicate that CSTC microcircuits lose this balanced state. Mutant huntingtin (mHTT) triggers both loss of inhibitory elements and pathological excitatory changes. The first line of evidence concerns altered GABAergic interneurons. HD pathology disproportionately affects fast-spiking (parvalbumin, PV+) interneurons. In HD mouse models, PV+ interneurons are significantly reduced in number [96], whereas other interneuron types (somatostatin-positive, calretinin-positive, cholinergic) are relatively spared (though cholinergic cell bodies are reduced in size) [96]. This loss of PV interneurons reduces the feed-forward inhibition normally exerted on MSNs. Paradoxically, remaining GABAergic interneurons—especially somatostatin-expressing “low-threshold spiking” interneurons—become pathologically active: optogenetic studies in Q175 HD mice show that silencing SOM interneurons markedly reduces the abnormally high frequency of spontaneous inhibitory postsynaptic currents (IPSCs) onto MSNs [97]. In other words, HD models develop a hyperactive SOM circuit that drives excessive GABA release onto MSNs, even as PV interneuron loss removes another inhibitory control. These findings suggest a reorganization of inhibition: PV-FSI loss diminishes one form of inhibition, while SOM interneurons aberrantly increase another.

The second line of evidence concerns glutamatergic synapses. HD has long been recognized as an excitotoxic disease. Cortical pyramidal neurons and thalamic inputs continue to provide glutamatergic drive to the striatum, but synaptic regulation is altered. HD MSNs show hyperexcitability: in vitro recordings from HD mouse cortex and striatum reveal increased spontaneous EPSCs onto projection neurons and diminished inhibitory currents [98]. In particular, Cummings et al. showed that in multiple HD mouse models, the frequency of spontaneous EPSCs is elevated while that of IPSCs is reduced [99], indicating net cortical disinhibition. This arises partly from reduced interneuron input (as above), but also from changes in glutamate receptor function (see below). Excess glutamate release and prolonged NMDA-receptor activation have been implicated in MSN death through classic excitotoxicity mechanisms [98].

The third line of evidence concerns NMDA/AMPA receptor function. The balance of NMDA vs. AMPA currents is altered in HD. Several HD models (e.g., R6/2) show changes in NMDA receptor subunit composition and gating, favoring calcium-permeable or extrasynaptic channels [100]. This amplifies excitatory signaling. The sustained activation of extrasynaptic NMDA receptors—normally tightly regulated—now drives neurotoxic cascades. AMPA receptors in HD neurons may also show altered “flip/flop” subunit expression (affecting desensitization) and reduced peak currents in developing mice [101]. Together, these receptor changes potentiate glutamate-induced excitation.

The fourth line of evidence concerns chloride homeostasis and GABA signaling. A key mechanism regulating inhibitory strength is the chloride equilibrium potential (E_GABA), set by cation–chloride cotransporters. In healthy neurons, the K^+^–Cl^−^ cotransporter KCC2 maintains low intracellular Cl^−^ concentrations, so that GABA_A currents are hyperpolarizing [102,103,104]. In HD, several studies report an upregulation of the Na^+^–K^+^–2Cl^−^ cotransporter NKCC1 and/or downregulation of KCC2. This causes a depolarizing shift of E_GABA (intracellular Cl^–^ rises) and weakens GABAergic inhibition [105]. For example, Hsu et al. found that striatal NKCC1 is elevated in R6/2 and knock-in HD mice, and that MSNs in R6/2 mice exhibit a depolarized GABA reversal potential. Pharmacologically blocking NKCC1 with bumetanide (an FDA-approved diuretic) restored hyperpolarizing GABA currents in HD neurons and rescued motor deficits in R6/2 mice [105].

The fifth line of evidence regards astrocytic dysfunction. Astrocytes normally clear excess glutamate and K^+^ from the extracellular space [106,107]. In HD, mHTT expression in astrocytes impairs these homeostatic functions. Notably, Tong et al. showed that striatal astrocytes in R6/2 and Q175 HD mice have reduced expression of the Kir4.1 K^+^ channel, leading to elevated extracellular K^+^ [108]. Elevated ambient K^+^ depolarizes MSNs and increases their firing. The viral re-expression of Kir4.1 in HD astrocytes restored K^+^ buffering, normalized MSN excitability, and ameliorated motor symptoms.

Collectively, these findings show a multi-faceted E/I imbalance in HD: loss of key GABAergic interneurons and potassium-buffering channels, combined with enhanced glutamate drive and impaired inhibitory signaling, tipping striatal and cortical circuits toward hyperactivity and aberrant inhibition.

Circuit-level E/I disruption in HD has clear electrophysiological signatures. In neuronal recordings, HD models exhibit cortical hyperexcitability [98]. As noted, spontaneous EPSC rates in HD cortical pyramidal cells are elevated while IPSC rates are diminished, indicating net excitation. Moreover, juvenile R6/2 cortical neurons in slices become epileptiform when GABA_A receptors are blocked; bath application of the GABA antagonist bicuculline elicits more frequent burst discharges in HD cortex than in controls [98]. These findings suggest that cortical neurons in HD are closer to the firing threshold and more prone to synchronized bursting, reflecting E/I imbalance.

At the level of EEG and oscillatory activity, HD patients exhibit clear abnormalities. Quantitative EEG studies report a slowing of cortical rhythms: manifest HD patients have increased slow-wave (delta/theta) power and diminished alpha power, along with a loss of the normal anterior-to-posterior alpha gradient. These EEG changes suggest an excess of slow oscillations and cortical disinhibition. Although high-frequency oscillations (beta/gamma) have not been systematically characterized in HD, by analogy to other basal ganglia disorders, it is plausible that abnormal synchrony (e.g., reduced beta coupling) occurs.

Alpha rhythms (approximately 8–13 Hz) are among the most prominent electrical oscillations of the human brain [109]. They are most evident in posterior regions—the classic occipital “alpha wave” seen with eyes closed in relaxed wakefulness [110]. Alpha oscillations have long been associated with an “idling” cortical state, but contemporary evidence demonstrates that they play active functional roles rather than constituting mere background noise [111]. Far from indicating “idling” alone, alpha oscillations are now understood to play critical roles in sensory processing, attention, and network coordination. One key function is sensory gating and inhibition: alpha activity actively suppresses irrelevant or distracting inputs, effectively inhibiting cortical processing in task-irrelevant regions [112]. For example, when attention is directed to one visual hemifield, alpha power increases in the contralateral occipital cortex for the ignored hemifield, presumably to gate out distraction. Conversely, alpha power decreases in regions processing attended stimuli, releasing those circuits from inhibition. In this way, alpha oscillations support selective attention by inhibiting neural representations outside the attentional focus [112].

Alpha oscillatory phase and magnitude have been linked to the timing of neural excitability, suggesting an “inhibition-timing” mechanism in which alpha rhythms create cyclical windows of suppression and permissiveness for neural firing [112]. This rhythmic pulsing may optimize information processing by coordinating when neurons fire relative to the alpha phase. Alpha rhythms are also implicated in large-scale cortical coordination and cognitive processes. During memory tasks and internal attention (e.g., mental imagery or retrieval), parieto-occipital alpha oscillations often increase, which has been interpreted as the brain “disengaging” from external inputs and facilitating internal processing—a phenomenon sometimes described as alpha-mediated functional inhibition [112]. At the network level, alpha oscillations can synchronize activity across distant cortical areas, effectively linking regions into functional assemblies. Indeed, alpha is regarded as a fundamental rhythm for top-down cognitive control, with distinct brain networks (e.g., frontoparietal networks) modulating alpha power and inter-regional phase synchrony to influence perception and behavior [111].

Alpha oscillations emerge from the coordinated activity of excitatory and inhibitory neurons in both thalamic and cortical circuits [113]. Thalamo-cortical loops are a major source of alpha rhythmicity: reciprocally connected thalamic relay cells and inhibitory neurons in the TRN can generate robust ~10 Hz oscillations even in the absence of changing sensory input [114]. A subset of TC relay neurons function as intrinsic pacemakers—often termed high-threshold bursting cells—that can rhythmically fire in the alpha range due to intrinsic membrane properties [115]. These neurons express a combination of ion channels that produce alternating hyperpolarization and rebound depolarization. In particular, the interplay between T-type calcium currents (low-threshold Ca^2+^) channels that activate upon release from hyperpolarization) and HCN channels endow these cells with a tendency to oscillate at ~8–12 Hz [115]. When a TC neuron is inhibited and hyperpolarized, T-type Ca^2+^ channels recover from inactivation; the subsequent release from inhibition allows a burst of Ca^2+^–driven action potentials, after which HCN-mediated current can help pace the next cycle. Individual thalamic pacemaker cells can thus generate alpha-frequency bursting intrinsically via this HCN–Ca^2+^ interplay [115].

Within the thalamus, these oscillations are synchronized and controlled by circuit interactions. The TRN—a shell of GABAergic neurons surrounding the thalamus [116]—provides inhibitory feedback to the relay cells [117]. TRN neurons themselves receive excitatory input from both cortex and thalamic relay cells [118]. This forms a feedback loop: a burst in a TC relay cell excites TRN interneurons, which then deliver a GABA-mediated inhibitory pulse back to the TC cells, hyperpolarizing them. The TC cells, after a ~100 ms inhibitory period, rebound into the next burst, which again drives the TRN, and so on [115]. In essence, the oscillation is sustained by reciprocal coupling: TRN neurons fire rhythmic inhibitory volleys that pace the relay cells, and relay cell bursts re-excite the TRN. Importantly, TRN cells also inhibit each other, which helps sculpt and limit the spread of synchrony, preventing runaway excitation and tuning the rhythm’s coherence. This intrathalamic network can produce ~10 Hz rhythms akin to sleep spindles or wakeful alpha, depending on the level of neuromodulation [115]. For example, cholinergic input from the brainstem and basal forebrain can depolarize TC cells and adjust their bursting mode, effectively modulating alpha rhythm amplitude and frequency. In fact, elevated acetylcholine is known to promote the thalamic alpha rhythm by influencing these channel dynamics [119,120].

While the thalamus provides a core pacemaker, the cortex is an active partner in alpha generation. Cortical pyramidal neurons (especially in layer V of the neocortex) send feedback excitatory projections to both thalamic relay cells and the TRN [114]. This corticothalamic feedback can synchronize and enhance the thalamic oscillation or modulate its frequency [110]. For instance, if the cortex is engaged in processing, it may desynchronize thalamic alpha by providing constant excitatory drive, whereas in a quiescent state, cortical feedback can help sustain the rhythmic loop. The cortex itself can also generate local alpha oscillations in certain conditions: networks of cortical excitatory neurons and inhibitory interneurons can resonate at alpha frequency [121]. Such cortical alpha may be especially relevant to “idle” sensory areas or during internal tasks (where thalamic input is not primary). Notably, the gain and timing of alpha oscillations are sensitive to the balance of excitation and inhibition. Increasing global inhibitory tone (for example, via benzodiazepines or barbiturates) tends to decrease alpha power but may also slow its frequency, reflecting longer durations of the inhibitory postsynaptic potential (IPSP) [122]. In summary, alpha generation requires tightly coordinated E/I interactions: thalamic circuits provide a rhythmic template via T-current and HCN-driven bursts paced by TRN inhibition, and cortical circuits reinforce and modulate this activity through feedback loops and local oscillatory dynamics. The result is the prominent ~10 Hz oscillation that underlies healthy sensory gating and attentional cycles.

What causes the diminution of alpha rhythms in HD at the cellular and circuit level? Converging evidence suggests that the excitation/inhibition imbalance known to occur in HD’s neural circuits is a major factor disrupting alpha generation. In HD, multiple neuropathological changes—including interneuron loss, altered neurotransmitter function, and glial dysfunction—lead to a disruption of the normal inhibitory-excitatory equilibrium. This imbalance likely impairs the thalamocortical and cortical network dynamics that sustain alpha oscillations. There are a couple of key HD-related changes that can mechanistically undermine alpha rhythmicity.

HD not only kills striatal projection neurons—it also affects cortical GABAergic interneurons that are critical for pacing network oscillations. Post-mortem analyses reveal significant cortical interneuron loss in HD, especially in certain regions. For example, in advanced HD cases with prominent neuropsychiatric symptoms, up to ~80% of parvalbumin-positive (PV) fast-spiking interneurons are lost in the anterior cingulate cortex [123]. Other interneuron subtypes are also depleted (e.g., calbindin- and calretinin-expressing cells), although the degree can vary by region and symptom profile [123]. In animal models, interestingly, PV interneurons can be relatively preserved early on, but somatostatin-positive (SOM) and vasoactive intestinal peptide (VIP) interneurons show dysfunction: R6/2 HD mice exhibit a selective reduction in SOM and VIP interneuron markers in the cortex [124]. Interneurons are the primary source of cortical inhibition and rhythmic timing—PV cells tightly synchronize cortical oscillations (especially beta/gamma) by rapid perisomatic inhibition [125]. The loss or dysfunction of these interneurons in HD means less precisely timed inhibition onto pyramidal neurons. This E/I shift (in favor of excitation) makes it difficult for networks to engage in coherent 8–12 Hz oscillations. Simply put, fewer inhibitory neurons (or impaired inhibitory firing) lead to weaker oscillatory “brakes”. The result is cortical circuits that are hyperexcitable and unable to sustain the delicate, periodic inhibitory pulses required for alpha rhythm generation. Interneuron loss thus likely underlies the desynchronization and excess slow activity in HD EEG. (Notably, interneuron deficits also contribute to clinical phenomena such as cortical disinhibition and cognitive impairment.).

Beyond interneuron counts, the efficacy of inhibition in HD is compromised by altered neuronal chloride homeostasis. As mentioned earlier, in mature neurons, the K^+^-Cl^−^ cotransporter KCC2 normally keeps intracellular Cl^−^ low, so that GABA_A receptor activation causes Cl^−^ influx (hyperpolarizing inhibition) [126]. HD models show a downregulation of KCC2 and upregulation of NKCC1, the transporter that pumps Cl^−^ into cells [127]. Consequently, the GABA_A reversal potential becomes less negative—GABA_A currents shift towards depolarization rather than hyperpolarization. Indeed, in HD mice (R6/2 and others), GABA_A responses become paradoxically excitatory due to increased intracellular Cl^−^ [127]. This has profound effects on network oscillations: if inhibitory synapses cannot effectively hyperpolarize neurons, they cannot generate the rebound spiking or phasic silences that underlie oscillatory cycles. Instead of synchronized inhibitory–excitatory cycles, networks may fall into tonic depolarization or irregular firing. Essentially, GABA loses its “braking” power. The end result is an E/I imbalance skewed toward excitation and noise. This mechanism likely contributes to the loss of alpha in HD: even if interneurons fire at 10 Hz, their postsynaptic effect may fail to produce the coordinated pauses in pyramidal activity. (It is notable that pharmacologically restoring chloride balance—e.g., using the NKCC1 blocker bumetanide to enhance KCC2 function—can normalize GABA signaling and improve network function in HD models [127], underscoring how critical this issue is).

HD is characterized by glutamate dysregulation and neuronal hyperexcitability. Cortical projection neurons in HD can become overactive due to loss of inhibitory inputs and intrinsic alterations. Furthermore, the striatal degeneration in HD removes a major inhibitory influence on the thalamus and cortex (via basal ganglia circuits), potentially leading to thalamic overactivity and excessive cortical excitation. This glutamatergic overdrive means that neurons are firing in a more continuous or noisy fashion rather than in coordinated bursts. High background excitation can overwhelm the delicately timed oscillatory interactions required for alpha. For instance, if thalamic relay cells are tonically depolarized by excess glutamate, they may exit the burst mode needed for alpha pacing and instead fire arrhythmically. Similarly, cortical pyramidal neurons under constant excitatory pressure will have a reduced ability to engage in synchronous 10 Hz oscillatory firing—their membrane potentials may not repolarize sufficiently to be modulated at that frequency. In essence, the signal-to-noise ratio drops, and synchronous rhythmic firing is disrupted. Experimental support for this comes from observations of epileptiform activity and increased spontaneous firing in HD models, consistent with network hyperexcitability. Without appropriate inhibitory gating, the cortex and thalamus may default to slower oscillations (delta/theta) or incoherent activity. Thus, the alpha attenuation in HD can be viewed as a consequence of runaway excitation overwhelming the normal oscillatory regime.

Glial cells—particularly astrocytes—help regulate E/I balance by clearing neurotransmitters and buffering extracellular ions. In HD, astrocytes are profoundly affected by mutant huntingtin. HD astrocytes show an impaired uptake of glutamate due to a reduced expression of glutamate transporters EAAT2/GLT-1 and GLAST [128]. Consequently, synaptic and extrasynaptic glutamate linger at higher concentrations, causing prolonged neuronal depolarization and overactivation of NMDA receptors [129]. This astrocytic failure contributes to excitotoxic stress on neurons and elevates baseline excitation (as neurons are less shielded from glutamate spillover). Additionally, HD astrocytes have impaired K^+^ buffering capacity—for example, they show reduced inward-rectifier K^+^ currents (Kir4.1 channels) [129]. During intense neural activity, extracellular K^+^ can accumulate if not properly taken up by astrocytes, leading to the depolarization of nearby neurons and increased excitability. Together, these astrocytic deficits tilt networks toward hyperexcitation and suppress the physiological conditions required for alpha oscillations. Normally, a burst of activity is followed by efficient neurotransmitter clearance and ion rebalancing, allowing neurons to recover and fire again rhythmically. In HD, glial dysfunction means that after a neuron fires, glutamate and K+ persist, causing neurons to remain depolarized or erratically firing rather than entering a synchronized silent phase. The net effect is a breakdown of the cyclical firing pattern that underpins alpha rhythms. Moreover, astrocyte dysfunction can directly alter inhibitory transmission: if astrocytes do not remove GABA or provide metabolic support to interneurons, inhibitory efficacy further declines.

Although the focus is often on the cortex, the subcortical circuit changes in HD likely contribute to the disruption of alpha rhythms. The striatum normally provides inhibitory gating of thalamocortical signals via the indirect pathway; in HD, early loss of striatal neurons leads to the disinhibition of the thalamus. A hyperactive or dysregulated thalamus may fire out of sync with cortical rhythms. Although HD pathology is less pronounced in the thalamus than in the striatum, some thalamic atrophy and neuronal dysfunction occur in later stages. If the population of thalamic pacemaker neurons or TRN neurons is compromised (or receives aberrant inputs), the thalamic contribution to alpha rhythmicity may diminish. Additionally, the loss of striatal GABAergic output and altered basal ganglia neurotransmission (e.g., dopamine, etc.) can induce cortical oscillatory changes. For example, increased oscillatory noise in motor circuits caused by basal ganglia dysfunction may manifest as beta/alpha disruptions and contribute to motor symptoms, such as chorea, via oscillatory disinhibition. HD thus produces a multilayered E/I disruption—from synaptic microcircuits to thalamocortical mesoscale networks—that collectively undermines the generation of stable alpha oscillations.

Large-scale imaging and pathology data confirm that HD disrupts E/I circuitry. Structural MRI shows early and severe atrophy of the dorsal striatum (caudate and putamen) as well as progressive cortical thinning in motor, frontal, and sensory regions in HD patients [130,131,132]. Studies by Vonsattel and DiFiglia and subsequent researchers show that striatal MSNs are preferentially lost, followed by degeneration of cortical pyramidal neurons [133]. PET imaging reveals hypometabolism in these regions, while diffusion MRI detects degeneration of corticostriatal white matter tracts even before clinical onset [134]. These macroscopic changes imply that excitatory projections (from cortex to striatum, and from thalamus to striatum/cortex) are progressively disrupted, thereby altering the dynamics of large-scale networks.

At the molecular level, proton MRS and histology reveal markers of E/I imbalance. MRS in HD mouse striatum and human striatum consistently shows decreased N-acetyl aspartate (NAA), a neuronal health marker, and increased myo-inositol (a glial marker), reflecting neuron loss and gliosis [135]. Glutamate and GABA levels are also altered: some studies report reduced striatal glutamate in manifest HD. Zarate et al. showed that GABA levels measured by MRS correlate with corticostriatal synapse density [135], suggesting that in HD, the decline of inhibitory synapses parallels neurochemical changes. Immunohistochemistry confirms the loss of PV interneurons in the striatum (as discussed above) and reduced GABA_A receptor binding [105]. Conversely, markers of excitatory transmission (e.g., vesicular glutamate transporters) become dysregulated as synapses degenerate. In summary, imaging and pathology reveal that HD targets both excitatory projection neurons and a subset of inhibitory interneurons, producing an imbalance in neurochemical markers of excitation and inhibition.

Conceptually, the E/I imbalance in HD parallels hypotheses proposed for other neuropsychiatric disorders, though the underlying causes differ. In autism spectrum disorder (ASD) and schizophrenia (SCZ), researchers have posited a similar microcircuit shift toward excitation. For example, Foss-Feig et al. suggest that an increased E/I ratio due to excess glutamatergic drive or impaired GABAergic function—underlies key features of both ASD and SCZ [136]. In ASD, this is supported by the high prevalence of epilepsy (reflecting cortical hyperexcitability) and in SCZ by the NMDA receptor hypofunction model, where NMDA antagonism reduces interneuron activity and causes cortical disinhibition. HD shares aspects of this pathophysiology: as in ASD [137], HD patients—especially those with juvenile-onset—can experience seizures [138]; as in SCZ, HD features interneuron pathology and NMDA-mediated excitotoxicity. Oscillatory abnormalities also overlap: HD, ASD, and SCZ exhibit disrupted gamma-band synchrony and reduced resting alpha activity, reflecting impaired E/I dynamics, as reviewed by Foss-Feig et al. and supported by HD EEG findings. However, there are clear differences. ASD and SCZ are primarily developmental disorders involving subtle synaptic mutations and early-life insults, whereas HD is a late-onset genetic neurodegeneration. The timing of E/I imbalance also differed: it emerges in childhood or adolescence in ASD/SCZ, but typically appears during adulthood in HD, as degeneration progresses. Moreover, HD involves a toxic gain-of-function polyglutamine protein that triggers neuron death, whereas SCZ and ASD involve complex multifactorial pathologies. Despite these differences, the cross-disorder similarity is noteworthy: whether due to developmental miswiring or neurodegeneration, CSTC circuits are vulnerable to similar forms of E/I imbalance. This convergence suggests that therapeutic strategies targeting E/I balance (e.g., modulating interneuron function) could have cross-diagnostic applications.

In summary, the attenuation of alpha rhythms in HD is mechanistically linked to widespread E/I imbalance. HD leads to a loss of inhibitory neurons, impaired GABAergic signaling (due to chloride dysregulation), excess excitatory glutamatergic activity, and glial support failure, collectively pushing neural circuits toward hyperexcitability and disorganization. Alpha oscillations require a finely tuned balance—enough excitation to generate rhythm and sufficient inhibition to shape it and prevent runaway activity. In HD, the failure of inhibitory “brakes” and synchronization mechanisms results in disorganized firing and a shift toward slower or irregular oscillations (delta/theta), replacing the typical 10 Hz rhythm.

The degradation of alpha rhythms in HD has important implications for symptoms and could offer pathways for intervention. Functionally, the loss of alpha oscillatory activity likely contributes to the cognitive and attentional deficits in HD. As discussed, alpha rhythms are instrumental in attention control, sensory gating, and information processing. When these oscillations are weakened, patients may experience impaired sensory gating and reduced concentration—essentially a neural failure to filter out distractions. This corresponds with clinical observations: HD patients often have trouble focusing, are easily distracted, and display cognitive slowing [20,139,140]. If the brain is unable to generate robust alpha-mediated inhibition, irrelevant sensory inputs may intrude and overwhelm processing, leading to distractibility and cognitive overload. Moreover, alpha oscillations help coordinate brain regions during task execution; their loss may result in disorganized or inefficient brain network activation, contributing to executive dysfunction and memory problems in HD. Cognitive impairment in HD also likely reflects impaired cortical processing due to disinhibition. Prefrontal and striatal circuits supporting attention, working memory, and executive function depend on finely tuned E/I balance [141]; HD-related hyperexcitability and interneuron loss may degrade signal-to-noise ratio. Indeed, EEG slowing (characterized by delta increase and alpha loss) correlates with cognitive decline in HD, mirroring findings in Alzheimer’s disease [142]. Neurodevelopmental factors may also play a role: although HD typically manifests in mid-adulthood, mutant huntingtin is expressed in the brain from early development. Cepeda et al. report subtle cortical maldevelopment and hyperexcitability in juvenile HD models [98]. For instance, juvenile R6/2 mice show cortical neuron hyperexcitability and dysplasia-like changes shortly after birth, which may impair neural wiring [143]. This raises the possibility that E/I imbalance in HD begins as a developmental synaptopathy that later progresses into full degeneration.

Cortical disinhibition in HD is also evident at the behavioral level. Patients frequently display impulsivity, irritability, and poor impulse control—in essence, a failure to inhibit inappropriate thoughts or actions [144]. A potential EEG correlate is reduced alpha power, as alpha activity is associated with inhibitory control [145]. Studies have shown that lower alpha power correlates with poorer cognitive performance in HD, including lower MMSE scores. This suggests that alpha reduction is not just an epiphenomenon but may be directly related to the extent of cognitive impairment. Similarly, motor disinhibition (such as chorea) may be exacerbated by the loss of normal oscillatory gating. In healthy motor systems, rhythms such as mu/alpha and beta help regulate the timing of movements and suppress inappropriate motion [146,147]. Desynchronized cortical activity in HD could therefore contribute to erratic motor output by failing to appropriately inhibit and time neuronal firing in motor circuits.

On a larger scale, the disappearance of alpha rhythms indicates a breakdown of coordinated, synchronous activity across brain networks. HD patients often exhibit hyperconnectivity and aberrant connectivity in fMRI studies, which some interpret as the brain’s compensatory attempt or as a loss of focused network interactions [148,149]. Notably, one study found that decreased alpha power in early-manifest HD was associated with increased functional connectivity across large-scale networks (default mode, corticostriatal, hippocampal). The authors suggested that this reflects a decrease in inhibitory control in those networks, leading to excessive, non-specific coupling. Normally, alpha activity shapes functional networks by pruning irrelevant connections; in its absence, networks may become over-connected in a maladaptive manner, reducing the segregation of brain systems. This desynchronization and abnormal coupling can degrade the precision of neural communication necessary for complex tasks, further contributing to cognitive decline.

From a translational perspective, the pronounced alpha-band changes in HD position EEG are a promising tool for biomarkers and therapeutic monitoring. EEG is relatively inexpensive, non-invasive, and suitable for longitudinal use. Reductions in alpha power and slowing of peak frequency may serve as objective biomarkers of disease progression. Researchers have applied machine learning to qEEG features (including alpha power and coherence) to distinguish HD gene carriers from controls, suggesting that EEG measures could help identify at-risk individuals or track disease even before symptoms appear. In clinical trials, EEG endpoints may help determine whether a therapy has neurophysiological effects. For example, if a given drug aims to rebalance neurotransmitters or slow neurodegeneration, an accompanying stabilization or improvement of the alpha rhythm (such as an increase in alpha power or a slowing of the delta increase) would be encouraging evidence of target engagement. Some therapeutic approaches, such as neural stem cell transplants or neurotrophic factors, aim to restore interneuron function; EEG could directly measure whether cortical oscillatory activity is rescued as a result.

The mechanistic link between alpha deficits and E/I imbalance also suggests potential interventions. One strategy is targeting chloride transporters. As noted, NKCC1 inhibition by bumetanide has shown promise: in HD mice, bumetanide shifted E_GABA toward more hyperpolarized values, reduced excitability, and improved motor and cognitive outcomes [105]. Blood-brain penetrant NKCC1 inhibitors or KCC2 enhancers could similarly restore inhibitory strength. A second approach involves astrocyte modulation. Enhancing Kir4.1 channel expression or function may support K^+^ homeostasis. In experimental HD models, viral delivery of Kir4.1 to striatal astrocytes normalized extracellular K^+^ and attenuated motor symptoms [108]. Drugs that boost astrocyte GLT-1 (e.g., ceftriaxone) may aid glutamate clearance, though clinical efficacy remains unproven [150,151,152]. A third therapeutic route may involve enhancing GABAergic signaling. The pharmacological activation of GABA_A receptors (benzodiazepines, neurosteroids) or GABA_B receptors (baclofen) might compensate for lost inhibition [153,154,155]. Alterations in GABA_A receptor subunits and benzodiazepine binding have been observed in HD brain tissue, suggesting remaining therapeutic targets. Small clinical trials of GABA agonists in HD (e.g., tiagabine, valproate) have shown limited benefit [156,157], but newer agents such as selective neurosteroids are being explored. A fourth possibility involves reducing glutamate excitability. NMDA receptor antagonists or modulators (e.g., memantine) may reduce excitotoxicity [158]. However, HD trials of memantine and amantadine in HD have produced mixed results, likely reflecting the challenge of reducing glutamate activity without overly suppressing function [159,160,161,162].

In addition to pharmacology, neuromodulation techniques may offer ways to enhance alpha rhythms. Transcranial alternating current stimulation (tACS) at alpha frequency (≈10 Hz) can entrain cortical oscillations [163]; in theory, applying 10 Hz tACS to the parietal or occipital cortex in HD patients might strengthen alpha activity and improve attention—though this remains speculative, it is supported by preliminary evidence from other populations. Neurofeedback training presents another possibility: patients could learn to increase alpha power via real-time EEG feedback, potentially enhancing their inhibitory control and cognitive focus. While still in the early stages, these approaches support the idea that alpha oscillations may be more than biomarkers—they could be direct therapeutic targets. If alpha activity can be safely enhanced in HD, some symptoms of cortical disinhibition might be ameliorated.

Finally, the link between alpha rhythms and cognition suggests that EEG alpha could serve as a readout for cognitive interventions. For example, if cognitive remediation or brain stimulation proves effective in HD, corresponding increases in task-related alpha modulation or normalization of EEG patterns may be expected. Likewise, lifestyle or symptomatic interventions (such as improved sleep or physical activity) might be reflected in healthier EEG profiles. In neurodegenerative diseases such as Alzheimer’s, alpha deficits are being investigated as biomarkers and therapeutic targets; although the underlying pathologies differ, HD appears to converge on a similar electrophysiological signature of neural disconnection and slowing. Thus, studying alpha oscillations in HD not only deepens the understanding of disease mechanisms—particularly E/I imbalance and network dysfunction—but also supports their translational value: quantitative EEG measures (especially alpha power and frequency) are candidate biomarkers for clinical trials, and therapies that restore the E/I balance may be guided and monitored through their effects on brain rhythmic activity.

## 6. Neurovascular Coupling in Huntington’s Disease—Primary Driver or Downstream Passenger?

Mounting evidence shows that the neurovascular unit (NVU) is perturbed in HD well before the classic striatal–cortical pathology dominates the clinical picture. However, whether this perturbation initiates neuronal stress or merely tracks it remains unsettled. At the cell-autonomous end of the spectrum, human iPSC–derived brain-microvascular endothelial cells carrying mutant HTT reveal intrinsically leaky tight junctions, blunted Wnt/β-catenin signaling, and aberrant angiogenic sprouting even in the absence of neurons [1]. Rodent studies reinforce an early-onset vascular phenotype: longitudinal arterial spin labeling and hypercapnic fMRI in zQ175DN knock-in mice reveal cortical hyperperfusion at three months—well before motor signs appear—followed by a progressive collapse of CO_2_ cerebrovascular reactivity (CVR) from six months onwards [2]. These findings indicate that mutant huntingtin can destabilize vascular tone and reactivity independently of large-scale neurodegeneration.

Human imaging studies reveal parallel abnormalities in presymptomatic carriers. Hypercapnic BOLD-fMRI shows globally blunted CVR and prolonged vascular time-to-peak in gene-expanded individuals who are still asymptomatic; the spatial distribution of delayed vascular response overlaps with dilated perivascular spaces on structural scans, hinting at early microangiopathy. Arterial spin labeling perfusion MRI demonstrates heterogeneous cortical CBF reductions—most pronounced in parietal and frontal cortices—that are not fully attributable to gray-matter atrophy. Importantly, lower CBF correlates with higher plasma neurofilament light and smaller caudate volumes, tying vascular insufficiency to molecular and structural markers of neuronal injury.

Electrophysiological evidence converges on a temporal uncoupling of neural and vascular oscillations. Simultaneous EEG–fNIRS recordings reveal that phase coherence between cortical alpha rhythms (8–12 Hz) and slow (~0.1 Hz) hemodynamic oscillations is already 17% lower in presymptomatic carriers and drops by one-third in early-manifest HD, whereas slow-wave EEG power actually increases. This pattern suggests that NVU synchrony deteriorates early and declines independently of absolute neural power, potentially undermining metabolic support precisely when cortical networks become more energetically vulnerable.

Taken together, these multi-scale observations support a two-hit model: HTT-intrinsic endothelial dysfunction primes the NVU, resulting in hyperperfusion and fragile CVR in the prodromal stage; as synaptic and metabolic stress escalates, the compromised vasculature fails to meet local demands, leading to regional hypoperfusion, impaired CO_2_ reactivity, and progressively weaker neurovascular phase-locking. Nevertheless, the correlations between CBF decline, neurofilament light, and caudate atrophy leave open an alternative interpretation—that vascular failure primarily mirrors neuronal degeneration rather than drives it.

Decisive tests will require (i) endothelial-specific mHTT knockdown or rescue in knock-in mice to determine whether restoring vascular gene expression delays electrophysiological slowing, and (ii) five-year multimodal follow-up of Enroll-HD sub-cohorts with annual ASL-CBF, hypercapnic CVR, and high-density EEG to assess whether baseline NVU metrics predict subsequent neuronal atrophy or vice versa. Until such causality is established, neurovascular coupling abnormalities should be interpreted as an early, disease-related co-pathology—potentially druggable but not yet proven to be the initiating event in HD pathogenesis.

## 7. Limitations and Future Directions

Although this review included 22 studies with a combined sample of over 500 HD patients, substantial variation across studies limits the interpretability and comparability of findings. The following are the main limitations and proposed directions for future studies.

### 7.1. Signal Acquisition and Pre-Processing

Signal acquisition and preprocessing remain the principal bottlenecks for comparability across Huntington’s disease EEG studies, largely because every laboratory still follows its own idiosyncratic workflow. Channel density spans nearly two orders of magnitude—from the 8-channel, 100 Hz amplifiers used in early clinical papers to modern 128-channel, 1 kHz high-density arrays—resulting in spatial sampling, spectral resolution, and the capacity to compute connectivity or microstates all differ in ways that cannot be reconciled retrospectively. A practical compromise is to mandate at least a 32-channel extended 10–20 layout (including Fz, FCz, Cz, Pz, Oz, and lateral fronto-temporal pads) for every new dataset. When high-density systems are available, a downsampled 32-channel surrogate should be stored alongside the full montage, allowing multi-site mega-analyses to rely on a harmonized core set.

Sampling rates and analog filter settings also vary widely: some laboratories constrain data to 0.3–35 Hz with 100 Hz digitization, precluding analysis of beta-2/low-gamma power and phase-lag estimates, while others sample at 2 kHz with DC coupling. Recording at ≥1 kHz with a hardware high-pass filter of ≤0.1 Hz and a low-pass of ≥250 Hz, followed by software-decimation to 500 Hz after anti-alias filtering, preserves the full physiologically relevant spectrum while keeping file sizes manageable. Because the reference montage affects all connectivity and source-localization metrics, raw data should be acquired against FCz or CPz but always re-referenced offline to a robust average after bad-channel interpolation, and both versions (raw and re-referenced) should be archived.

Basic amplifier hygiene matters too: skin–electrode impedance must remain below 10 kΩ, the amplifier should be DC-coupled with an input impedance above 1 GΩ, and the precise hardware high-pass pole (e.g., 0.1 Hz, −3 dB) has to be documented. Artifact management needs to move from purely manual trimming—still common in several HD studies—to fully logged, reproducible pipelines. A concrete template includes: initial 0.1–100 Hz band-pass filtering and notch filtering; automated channel rejection (variance, flat-line, line-noise); spline interpolation; ICA or SOBI on a 1–45 Hz copy with ocular, muscle, and ECG components labeled by ICLabel (at ≥0.8 probability) and removed; residual high-frequency muscle artifact suppressed with algorithms such as Zapline-plus; and segment rejection triggered when joint probability exceeds five standard deviations or RMS power spikes indicate head movement. Every decision point should be logged in a JSON sidecar using BIDS-EEG conventions so that other groups can replay or modify the pipeline.

Epoching requires vigilance control: five continuous minutes of eyes-closed wakefulness, monitored by simultaneous EOG, EMG, and video, should yield at least 60 non-overlapping, artifact-free four-second epochs after discarding segments containing slow EOG drift (>50 μV) or muscle bursts (>25 Hz). Spectral analysis should follow a standardized Welch routine—four-second Hanning windows, 50% overlap, 0.25 Hz bins—reporting absolute power in μV^2^/Hz, relative power as a percentage of the 2–45 Hz band, or log-ratio formulation log[p/(1 − p)], with explicit declaration of which metric was used.

True reproducibility also depends on transparent data sharing: raw, cleaned, and derivative files—together with amplifier model, filter settings, electrode layout, posture, time-of-day, medication status, and recent caffeine or nicotine intake—should be uploaded to an open repository such as OpenNeuro or DANDI, in full BIDS compliance. Finally, each site should run an annual phantom sine-wave test (10 μV, 10 Hz) to confirm frequency response within ±0.5 dB and record a one-minute calibration (eyes-open then eyes-closed) for every participant to verify the detectability of the individual alpha peak across laboratories. If the field converges on these exact, auditable standards, variance stemming from hardware idiosyncrasies or ad-hoc cleaning choices will collapse, allowing genuine disease-related electrophysiological signatures to emerge with effect sizes large enough to support regulatory-grade biomarker development.

### 7.2. Biological Confounders

Even the most technically rigorous EEG protocol can yield misleading results if biological confounders are not accounted for—three of which are especially pervasive in HD research. First, medication effects. Medication is ubiquitous in HD: antipsychotics are commonly prescribed to manage chorea and irritability, SSRIs or SNRIs for mood disturbances, benzodiazepines for insomnia or anxiety, and VMAT-2 inhibitors such as tetrabenazine or deutetrabenazine to suppress motor symptoms [164,165,166,167]. These drugs are known to slow dominant frequency, suppress beta power, and disrupt thalamocortical coherence [168,169,170,171,172,173]. Only a small number of studies include drug-free subgroups or report dosage details, making it difficult to separate pharmacological effects from disease-related signals. Consequently, genuine disease-related slowing is frequently conflated with pharmacological sedation, leaving the field uncertain whether an observed delta increase or beta2 surge reflects neurodegeneration or medication effects.

This confounding is far-reaching. Because pre-manifest carriers are typically unmedicated while early-manifest patients often receive neuroleptic treatment at diagnosis, apparent group differences may reflect treatment status rather than a true stage of illness. For instance, quetiapine or risperidone can raise absolute delta power by 30–50%, masking the subtler (~15%) slowing reported in unmedicated cohorts. Similarly, benzodiazepine-induced high-beta peaks closely resemble the “compensatory” beta2 elevations sometimes interpreted as cortical hyperexcitability. At the network level, GABAergic medications shorten phase-locking times, artificially reducing weighted phase-lag indices and inflating graph-theoretical path length—metrics now championed as preclinical HD biomarkers.

There are practical strategies to prevent pharmacological noise from being interpreted as pathophysiology. When ethically feasible, investigators should include a medication-naïve stratum—carriers with CAP > 250 but UHDRS-TMS ≤ 5—and, where withdrawal is impossible, recruit gene-negative relatives on matching regimens to isolate drug effects. All psychoactive agents should be documented using the WHO-ATC classification system, with details on dose, formulation, half-life, time since last administration, and—for antipsychotics and benzodiazepines—conversion to chlorpromazine or diazepam equivalents. Plasma-available load can then be modeled as a continuous covariate, for example by multiplying dose by the drug’s elimination constant, and primary analyses should be repeated with and without this adjustment.

Sensitivity analysis should exclude participants who have taken GABAergic sedatives within the past 36 h, those on polypharmacy (more than two CNS-active agents), and those receiving high-potency dopamine antagonists, to ensure that findings are not driven by a pharmacologically distinct subgroup. In longitudinal studies, clinically necessary medication adjustments should be randomized or at least scheduled, converting them into experimental variables rather than uncontrolled confounds. Finally, researchers should collect auxiliary physiological signals such as ECG, pupillometry, or wrist-actigraphy as objective proxies for sedation and autonomic state and statistically control for these in EEG analyses using joint ICA or hierarchical regression models.

Only by embedding such methodological controls can the field generate EEG-derived effect-size estimates that are robust enough to support regulatory qualification of biomarkers in HD.

Second, sleep and circadian disruption. HD gene carriers commonly experience progressive insomnia, fragmented REM sleep, excessive daytime sleepiness, and dampened melatonin rhythms. As a result, “eyes-closed rest” is not a stable or uniform state, and between-group differences in alpha or theta activity may simply reflect differences in arousal. Yet beyond dedicated PSG studies, sleep quality is rarely assessed beyond subjective self-report scales. Third, systemic and neurovascular factors. Weight loss, autonomic dysregulation, endothelial dysfunction, and impaired neurovascular coupling emerge early in HD and may distort cortical oscillations through metabolic or hemodynamic effects. Only two studies have paired EEG with fNIRS or cardiovascular monitoring, leaving the broader field largely blind to these influences.

Future investigations should therefore (i) include medication-naïve or wash-out subgroups where ethically possible, or at minimum, record full pharmacological data for uses as covariates; (ii) incorporate objective sleep assessments—such as actigraphy, melatonin assays, or concurrent PSG—to quantify vigilance state rather than assume it; and (iii) integrate cardio-metabolic and neurovascular readouts (e.g., heart-rate and blood-pressure variability, fNIRS, transcranial Doppler) to control for systemic influences and examine how cortical rhythms relate to vascular health across disease progression.

### 7.3. Small Sample Sizes

Small sample size remains the most pervasive—and underacknowledged—threat to the credibility of EEG findings in HD. A survey of the literature shows a median cohort of just twenty-four mutation carriers, and fully one-third of studies analyzed fewer than fifteen. At this scale, a typical mid-range disease effect—Hedge’s *g* around 0.5 for alpha or theta power—would require approximately sixty-four participants in each arm to reach the conventional 80% power at an α of 0.05, while subtler network-level changes closer to g = 0.3 would demand more than 175 per group. Undersized experiments therefore suffer a dual penalty: they are likely to miss true effects (type II error), and when they yield statistically significant findings, those estimates are almost certainly inflated by winner’s curse bias, destabilizing any subsequent meta-analyses.

Solving this small-*n* problem will require structural, not incremental, change. The most direct solution is to embed EEG protocols within large-scale observational platforms such as Enroll-HD or HDClarity, using harmonized acquisition protocols that enable each site to contribute roughly twenty-five carriers per disease stage annually. Across ten international centers, this would generate the 250–300 participants needed for biomarker-grade discovery. Sequential trial designs can further increase efficiency: investigators may pre-register a maximum sample size—say, 200 carriers—but analyze interim batches of 30 participants while using an alpha-spending rule (e.g., O’Brien-Fleming or Pocock boundaries), halting data collection early only if effect estimates stabilize. Longitudinal within-subject designs also boost statistical power: three-wave designs (baseline, 18 months, 36 months) treat each participant as their own control, making changes with *g* = 0.3 detectable with roughly forty-five carriers when the intra-individual correlation exceeds 0.6.

As data accumulate across sites, Bayesian hierarchical models can integrate estimates across stages and centers, shrinking outliers toward the population mean and yielding credible intervals that remain informative even when individual strata are small. Finally, mandatory deposition of raw EEG and harmonized phenotypic tables in BIDS-compliant repositories will also enable living meta-analyses, where every new dataset is automatically folded into a continuously updated effect-size estimate, so the field no longer has to wait for a single mega-trial to converge on stable numbers. Only by institutionalizing these consortium-based, sequential, longitudinal, and Bayesian approaches can HD EEG research accumulate the statistical mass required for regulatory-grade biomarker qualification.

### 7.4. Multiple Comparisons

Failure to control the family-wise error rate is a quiet but pervasive weakness in the HD-EEG literature. Nearly every dataset is parsed across three axes—nineteen or more scalp sites (or thousands of LORETA voxels), six to eight canonical frequency bands, and often dozens of behavioral covariates—yet many studies still apply uncorrected *p* < 0.05 thresholds or, at best, Bonferroni corrections within isolated domains. The result is an abundance of false-positive “hotspots” that cannot be replicated: a frontal theta increase in one study, a parietal alpha split in another—each surviving only because the hundreds of concurrent tests that should dilute statistical significance are ignored. The problem is most acute in voxelwise source analyses, where up to 30,000 *t*-tests are sometimes interpreted with nothing more than a nominal *p* < 0.05 cutoff. Even studies citing “FDR correction” rarely clarify whether they used the Benjamini–Hochberg, Benjamini–Yekutieli, or Storey’s *q*-value procedure—distinctions that matter when spatial autocorrelations are high, as they are in EEG data. Some groups try to circumvent this issue by collapsing across all electrodes, but this merely trades spatial precision for uncontrolled increases in temporal and spectral comparisons.

The cure is methodological discipline. Before data collection begins, investigators must pre-register the full set of statistical comparisons they intend to run—ideally confined to a handful of anatomically or functionally defined regions of interest—and commit to a correction method suited to EEG’s spatial and spectral structure. Cluster-based permutation testing remains the gold standard, as it accounts for data smoothness: by shuffling labels a thousand times and computing the maximum cluster statistic each time, one obtains an empirical null distribution that automatically adjusts for the fact that neighboring electrodes and adjacent frequencies move together. Where permutation testing is impractical, a Benjamini–Yekutieli FDR with its built-in correction for positive dependency is preferable to the more permissive Benjamini–Hochberg method common in earlier HD work. If voxelwise source maps are necessary, random field theory can provide a parametric alternative—but thresholds should be reported as corrected *p*-values (e.g., *p* < 0.05 FWE) rather than “uncorrected with cluster extent > 100 voxels,” a loophole through which many spurious effects enter the literature.

Equally crucial is full transparency in reporting. Authors should list the total number of statistical tests performed, the exact correction algorithm, any α-spending plan for interim analyses, and provide full tables of both corrected and uncorrected *p*-values along with effect sizes and confidence intervals. Sharing the analysis code and permutation seeds in a public repository allows others to reproduce the thresholding pipeline verbatim. Finally, journals and reviewers must stop accepting topographic plots labeled simply with “*p* < 0.05” unless the figure legend specifies, for example, “corrected for N = 684 comparisons”. If such standards are enforced, the patchwork of inconsistent EEG abnormalities currently reported in HD will likely consolidate into a smaller set of robust, replicable effects.

### 7.5. Effect Size Reporting

A second, subtler weakness of the HD-EEG literature is the inconsistent and often arbitrary reporting of effect sizes. Approximately half of all studies still stop at *p*-values, a quarter provide group means with standard deviations but omit standardized statistics, and the remainder employ a mixture of Cohen’s *d*, Hedge’s *g*, η^2^, or percentage change—often without specifying the conventions used or how the values were derived. This inconsistency makes it impossible to determine whether a reported delta-power increase is a clinically meaningful shift or a trivial fluctuation exaggerated by sampling noise. As regulatory agencies and health-technology assessors now require magnitude-of-benefit estimates—not merely statistical significance—HD neurophysiology risks marginalization unless reporting practices are improved.

Moving forward, every EEG study in HD must treat effect size as a primary outcome. For between-group comparisons, the default should be Hedge’s *g* (corrected for small-sample bias), with authors required to report the point estimate, its 95% confidence interval, and—preferably—a Bayesian credible interval to convey both precision and evidential strength. For repeated-measures or mixed-effects models, partial ω^2^ is recommended, as it quantifies the variance uniquely attributable to disease status while adjusting for participant and site effects; η^2^, which is sensitive to model complexity, should be avoided. Connectivity graphs require a slightly different vocabulary—Fisher-*z*-transformed r for edge weights and Cohen’s q for between-group differences—while microstate or spectral-peak timing benefits from the non-parametric Cliff’s δ when distributions are skewed.

Crucially, studies must predefine what constitutes a minimal clinically important difference. For example, a 0.4-SD slowing of individual alpha frequency has been linked to approximately six months of functional decline on the Total Functional Capacity scale, offering a concrete threshold for prognostic utility.

Consistent and transparent effect-size reporting offers three key advantages. First, it enables power calculations based on empirical evidence rather than guesswork; if the literature converges on Hedge’s *g* ≈ 0.45 for alpha suppression in premanifest HD, then multicenter trials can plan for ~140 carriers per arm to achieve 90% power. Second, it facilitates meta-analysis without the need for ad-hoc conversions or contacting authors for raw data. Third, it allows clinicians to translate electrophysiological findings into meaningful clinical terms: a 1-SD drop in alpha-theta border power has been linked to a 2-point rise in UHDRS motor scores over 18 months—an outcome far more actionable than *p* = 0.003.

The practical solution is straightforward. Journals should mandate an “Effect Size Methods” subsection detailing the chosen metric, calculation formula, software version (e.g., esc or effectsize in R), and the raw numbers used. Preprint servers can flag manuscripts that omit standardized magnitudes, and peer reviewers should insist on confidence intervals alongside every *p*-value in tables and figures. Once these practices become standard, the field will be equipped not only to assess whether HD EEG findings are statistically valid, but also whether they are clinically meaningful.

### 7.6. Possible Publication Bias

Publication bias is the most invisible, yet arguably the most corrosive, threat to the credibility of EEG research in HD. A glance at the literature reveals a striking asymmetry: papers announcing “significant” alpha slowing or connectivity hypersynchrony pass review with ease, while null or equivocal findings virtually rarely reach publication. This imbalance is amplified by the field’s small-sample culture—when a study with fifteen carriers fails to replicate a previously reported theta surge, authors often attribute the result to low power and quietly archive the dataset. Conference abstracts compound the problem: preliminary posters routinely showcase eye-catching topoplots, but follow-up publications materialize only if the effects withstand further scrutiny—which they often do not. Over time, citation cascades inflate the perceived robustness of early positive findings, creating a self-reinforcing illusion that certain EEG signatures are “established,” even though the underlying evidence base is highly selective.

The downstream damage is substantial. Meta-analyses built on a skewed corpus overestimate effect sizes and feed them back into power calculations, leading to trials that appear adequately powered on paper but are doomed to fall short in practice. Clinical scientists, attracted by seemingly strong biomarkers, embark on costly multimodal research only to discover that the promised EEG signals fail to replicate under rigorous control conditions. Therapeutic programs may then dismiss EEG altogether as unreliable, slowing translation at a time when electrophysiological measures could provide valuable complements to imaging and fluid biomarkers.

Structural reforms are required to correct this imbalance. Every HD EEG study should be preregistered on an open platform such as OSF or ClinicalTrials.gov, with primary and secondary endpoints—and analysis plans specified in advance. Negative or null outcomes should be uploaded as citable preprints within a fixed timeframe (for example, twelve months after data freeze), regardless of journal acceptance, ensuring they enter the scholarly record and future meta-analyses. Journals should expand the use of Registered Reports, in which peer review occurs before results are known and publication is guaranteed if the protocol is approved. This removes the incentive to chase statistical significance. Funding agencies should formally recognize the value of “null” datasets and reward investigators who contribute to a comprehensive and unbiased evidence base. Finally, the field would benefit from a centralized, continuously updated evidence repository—modeled after CAMARADES in stroke research—that aggregates raw EEG and phenotype data, updates effect-size estimates in real time, and displays funnel plots with live Egger tests to monitor asymmetry.

Only when negative and positive findings are given equal weight will HD electrophysiology escape the distorting effects of selective reporting and evolve into a genuinely predictive and clinically credible biomarker field.

### 7.7. No Validated Cutoff Values for Clinical Decision-Making

Despite five decades of EEG research in HD, not a single study has delivered a prospectively validated cutoff that clinicians can apply to an individual EEG trace—analogous to how neurologists use a UPDRS motor score ≥ 26 for Parkinson’s diagnosis or cardiologists flag a QTc > 500 ms for torsades risk. Most studies stop at reporting group mean differences—“alpha power was lower in HD (*p* = 0.004)”—without converting those statistics into clinically usable thresholds. When cut-points are proposed, they are typically derived post-hoc from the same dataset that established the effect and are rarely tested in an independent cohort, let alone prospectively. As a result, electrophysiologists presented with a single premanifest carrier’s EEG still have no evidence-based answer to the question: “Is this record normal or abnormal, and what is the probability of conversion within five years?”

Establishing actionable thresholds demands a workflow modeled on best practices from cardiology and oncology. First, investigators must predefine candidate metrics—such as the 7–9 Hz/4–5 Hz power ratio or a composite microstate instability score—and lock analytic settings before accessing outcome data. Second, discovery and validation datasets must be truly independent: derive the cut-off that yields the optimal Youden index or 95% sensitivity in a training sample, then freeze it and apply it unchanged to a geographically distinct replication cohort. Third, performance must be reported with full operating-characteristic curves, including sensitivity, specificity, positive/negative predictive value, and, crucially, calibration—the agreement between predicted and observed risk across deciles. Fourth, proposed thresholds must be stress-tested for robustness to medication effects, vigilance state, and equipment differences. Finally, the field must conduct prospective, longitudinal studies in which baseline EEG is used—blinded to outcome assessors—to forecast predefined milestones such as UDHRS motor conversion or a two-point decline in Total Functional Capacity. Only cutoffs that meet this rigorous validation pipeline should be adopted in clinical guidelines, with periodic recalibration as more diverse datasets become available.

Until such validated thresholds exist, HD EEG will remain a promising research tool but fall short of clinical utility. Developing and validating these cutoffs is therefore the critical next milestone in transforming EEG from a group-level observation instrument into a practical tool for individual-level decision support.

### 7.8. Limited Longitudinal Data to Establish Predictive Utility

Long-term prognostic value remains the missing piece in HD EEG research because the vast majority of datasets are single snapshots obtained at variable distances from clinical onset. Only a handful of studies—chiefly the PET-linked sleep work of the late 1990s and a few modern add-ons to Enroll-HD—track the same individuals beyond 12 months, and almost none are powered to relate baseline oscillatory metrics to hard outcomes such as diagnostic conversion or two-point decline in Total Functional Capacity. As a result, although group-level analyses show that premanifest carriers often show subtle alpha-theta border power, it remains unclear whether an individual with a 0.6-SD reduction in 8–9 Hz power is at 5% or 50% higher risk of converting within 5 years. Without longitudinal anchors, EEG cannot yet compete with volumetric MRI or plasma neurofilament light as a predictor of disease trajectory.

Moving from cross-sectional to predictive utility demands a re-engineered study architecture. First, EEG recordings should be integrated into the scheduled annual or biennial visits already mandated by Enroll-HD and HDClarity, ensuring at least three time-points per participant over a minimum of five years. This enables a shift from between-subject differences to within-subject analyses, which are significantly more statistically efficient. Second, quarterly at-home recordings using validated 16-channel dry-electrode headsets should complement clinic visits; early pilot studies suggest that 10-min resting-state EEGs are feasible and yield usable spectra once artifact pipelines are automated. Third, joint modeling frameworks should be adopted in which time-varying EEG features and survival-style conversion endpoints are estimated simultaneously, yielding dynamic risk scores that update whenever a new trace is added. Fourth, all cohorts should report a harmonized set of longitudinal outcomes—motor onset (DCL ≥ 4), annualized change in UHDRS-TMS, and loss of functional independence (TFC ≤ 10)—to allow pooled analysis across countries. Fifth, analysis plans must be pre-registered, explicitly detailing how missing data, medication changes, and hardware upgrades will be addressed, and interim de-identified datasets should be released regularly to allow continuous algorithm refinement by the research community.

Only through such multi-wave, federated, and openly shared designs will the field accumulate the empirical weight required to transform EEG from a descriptive marker of current status into a bona fide predictor of the individual disease trajectory. Until then, claims about prognostic utility will remain speculative, and clinical EEG will continue to play a secondary role to imaging and fluid biomarkers in trials and clinical monitoring.

### 7.9. Unclear Relationship to Meaningful Outcomes (Conversion, Functional Decline, Quality of Life)

The ultimate test of any biomarker is its ability to track or predict outcomes that matter to patients and clinicians—namely, disease conversion, loss of functional independence, and decline in the quality of life. Yet most HD EEG studies stop at correlations with intermediate measures such as UDHRS motor scores or isolated cognitive tests. Very few link electrophysiology to hard endpoints, and those that offer only cross-sectional snapshots: diminished alpha power is “associated” with lower MMSE; microstate class-A dominance “correlates” with higher UHDRS-TMS; lagged phase-synchrony “relates” to slower Symbol Digit performance. None of these findings tells a clinician whether a given EEG pattern predicts that a presymptomatic carrier will cross the diagnostic threshold within three years, lose two points on Total Functional Capacity in 18 months, or experience a clinically meaningful drop in self-reported well-being. Even large multimodal cohorts such as Enroll-HD rarely integrate electrophysiology with the patient-reported outcome measures (HD-QoL, EQ-5D) now required by regulators.

Closing this gap demands study designs explicitly aligned with patient-centered endpoints. First, every EEG protocol should define a composite outcome hierarchy in advance: (i) time-to-motor-conversion (DCL ≥ 4); (ii) annualized decline in TFC and HD-QoL; (iii) threshold events such as loss of driving license or employment. Second, analytic pipelines must convert EEG features into quantifiable risk metrics—e.g., “each 0.1-μV^2^/Hz drop in 8–9 Hz power increases the hazard of conversion by 8% (95% CI 4–12%)”—so clinicians can interpret them alongside imaging, genetics, and fluid biomarkers. Third, minimal clinically important differences must be predefined: for example, a 0.4-SD slowing in alpha frequency might equate to the functional impact of one UHDRS-TMS point per year and could thus inform trial endpoints. Fourth, patient-reported outcomes must be collected in tandem with EEG to allow models to determine whether EEG changes precede or merely reflect declines in mood, sleep, or everyday task efficiency. Finally, validation studies blinded to outcomes—must confirm that EEG to existing prediction algorithms materially improves calibration and net re-classification.

Until HD-EEG studies are explicitly tied to conversion, functional loss, and quality-of-life trajectories, their clinical relevance will remain theoretical. Building that bridge—from oscillations on a monitor to decisions in the consulting room—is the next critical frontier for the field.

### 7.10. Volume Conduction Issues

Volume-conduction remains the elephant in the room in HD EEG research. Although most connectivity studies acknowledge the problem in passing, many still rely on zero-lag metrics—such as basic coherence, Pearson correlations of power envelopes, or uncorrected amplitude coupling—that are mathematically guaranteed to reproduce scalp field spread. Because HD is dominated by widespread cortical slowing, nearby electrodes often detect the same large-scale oscillation with little or no phase delay. As a result, zero-lag measures tend to report spurious “hypersynchrony,” especially in delta and alpha bands. This problem is amplified by inconsistent referencing schemes and low-density montages: common-average or linked-ear references diffuse activity across channels, while 16-channel arrays lack the spatial resolution needed for reliable Laplacian transforms or beamforming.

Mitigation strategies are available but underutilized. First, all connectivity analyses should prioritize metrics that ignore instantaneous correlations, such as imaginary coherence, phase-lag index (PLI), weighted PLI, or orthogonalized amplitude envelope correlation as used in MEG. Second, sensor-level work ought to apply a surface Laplacian (e.g., spherical spline or CSD transform) to attenuate volume-conduction before any coupling metric is computed; this step alone removes up to 70% of spurious coherence in simulation. Third, researchers should favor source-space connectivity via spatial filters that minimize leakage, such as minimum-variance beam-formers (e.g., DICS) or leakage-corrected eLORETA, and then apply leakage-robust metrics on the reconstructed time series. Fourth, statistical significance must be benchmarked against surrogate datasets—such as time-shuffled or trial-shuffled surrogates—to ensure that observed effects exceed what volume conduction alone could predict. Finally, all studies must report coupling as a function of phase-lag; if 90% of significant edges cluster at 0 ± 5°, they are almost certainly artifactual and should be discarded or, at minimum, interpreted as “field-spread correlations” rather than “neural synchrony.”

Until HD-EEG studies adopt these basic safeguards, the literature will continue to mistake shared generators for genuine inter-regional coupling, inflating claims of compensatory hyperconnectivity and obscuring the subtler, time-lagged interactions that likely carry true pathophysiological significance.

### 7.11. Disentangling Oscillatory Peaks from Aperiodic (1/f) Spectral Tilt

One final and frequently overlooked confound in HD EEG analysis is the failure to distinguish genuine oscillatory activity from the aperiodic 1/f spectral background. Conventional EEG analyses typically treat the power spectrum as a series of discrete frequency bands over a flat baseline, interpreting increases in delta power or a decrease in beta power as changes in oscillatory strength. In reality, the EEG spectrum comprises both periodic peaks and an aperiodic, scale-free component whose slope steepens when excitation–inhibition balance is altered—a hallmark of HD-related synaptic dysfunction. A steeper 1/f slope automatically inflates low-frequency power and suppresses high-frequency power, even if the underlying oscillators are unchanged. As a result, many “delta increases” and “alpha losses” reported in earlier HD papers likely reflect broadband spectral tilt rather than true rhythmogenesis.

Recent studies that fit and subtract the aperiodic baseline with different tools show that once the 1/f imprint is removed, several previously “significant” delta findings disappear, while genuine narrow peaks—most notably the 7–9 Hz alpha–theta border—persist, albeit with smaller effect sizes. Going forward, every HD-EEG analysis should fit the aperiodic slope and offset before computing band power, report the slope and intercept parameters alongside residual periodic estimates, and base connectivity on the cleaned oscillatory signal to avoid broadband-driven artifacts. Where possible, legacy datasets should be reanalyzed using modern decomposition methods to distinguish rhythm-specific pathology from global spectral steepening, enabling more precise linkage between electrophysiological markers and precise cortical circuit dysfunction.

### 7.12. Emerging Directions

Several intersecting developments in technology and basic science are now enabling a shift from descriptive electrophysiology toward mechanistic insight and intervention in HD. First, multimodal convergence. The combination of HD-EEG-MEG, ultra-high-field MR spectroscopic imaging of GABA and glutamate, and diffusion-weighted tractography is becoming increasingly feasible. These multimodal datasets allow researchers to map how the loss of striatal GABAergic tone disrupts thalamocortical circuits, alters oscillatory timing, and reshapes large-scale network topology.

Second, portable neuroscience. The advent of dry-electrode headsets, ear-EEG devices, and camera-based photoplethysmographic proxies for alpha rhythm enables multi-night, in-home monitoring with minimal participant burden. Such ecological sampling can reveal how fluctuations in stress, sleep, and medication adherence modulate cortical excitability—dynamics that remain inaccessible in traditional one-time recordings.

Third, closed-loop neuromodulation. Adaptive systems using transcranial alternating-current stimulation (tACS) and deep-brain stimulation (DBS) now allow for real-time detection of brain-state dynamics and the delivery of phase-locked stimulation. Proof-of-concept studies in Parkinson’s disease have shown that suppressing pathological beta bursts can improve bradykinesia; similar approaches could be applied in HD to target the 7–9 Hz deficits or delta hypersynchrony, offering a way to test both causality and therapeutic efficacy.

Fourth, neurovascular coupling as a treatment target. Techniques such as EEG–fNIRS and functional ultrasound imaging reveal that impairments in NVU function may precede large-scale neuronal changes. Another promising approach is neurofeedback, where patients learn to self-regulate brain activity by receiving real-time feedback from a brain-computer interface. EEG abnormalities in HD, particularly alpha power deficits, present an ideal target for neurofeedback training. In this method, the patient is rewarded for increasing or decreasing specific frequency bands, guided by audiovisual cues. Although no studies to date have applied neurofeedback in HD, alpha enhancement protocols based on the findings of this review represent a rational next step.

Fifth, genetics-informed modeling. Beyond CAG length, common SNP modifiers such as FAN1 and MSH3, along with somatic expansion rates, explain substantial variance in clinical onset. Incorporating these variables into mixed-effects models alongside electrophysiological data may improve personalized risk prediction and help identify gene-specific oscillatory patterns.

Finally, trustworthy AI pipelines. Foundation models trained on vast amounts of unlabeled EEG data can provide self-supervised embeddings that generalize across devices and sites. Combined with federated learning approaches, these models could enable secure, privacy-preserving analysis of global HD datasets and support the development of robust, reusable EEG biomarkers suitable for clinical and regulatory use.

Together, these innovations signal a future in which electrophysiological monitoring in HD is continuous, mechanistically informative, and directly coupled to adaptive therapeutic strategies—transforming the field from reactive symptom tracking to proactive, precision intervention.

## 8. Conclusions

Over five decades of research—ranging from analog eight-channel recordings to advanced, multimodal high-density arrays—a consistent electrophysiological signature of Huntington’s disease has emerged. Central to this pattern is a selective, progressive reduction in low-alpha (7–9 Hz) oscillations, detectable 10–15 years before motor onset, expanding to widespread cortical alpha loss as symptoms develop, and ultimately giving way to slow-wave dominance and low-voltage patterns in advanced disease. This spectral progression mirrors the anatomical spread of mutant huntingtin pathology through cortico-striato-thalamocortical circuits and correlates with CAG repeat length, clinical measures, and cognitive performance. Crucially, EEG abnormalities extend beyond changes in spectral power. Source-localized analyses show early disruptions in long-range alpha synchrony, accompanied by maladaptive delta and high-beta hypersynchrony in premotor and sensorimotor regions. Changes in EEG microstates (increased A/B, decreased C/D), rising signal complexity, and neurovascular uncoupling complete a multidimensional picture of network-level disintegration observable during wakefulness, task engagement, and sleep. These findings meet established biomarker criteria: they are early, scalable, biologically plausible, and functionally meaningful.

Nevertheless, translation to clinical practice remains limited. Most studies rely on small, cross-sectional cohorts, variable EEG hardware, and inconsistent preprocessing, all of which hinder replication. Medication use, sleep disruption, and systemic comorbidities further confound interpretation. Crucially, no EEG marker has yet been validated against hard outcomes such as phenoconversion or loss of functional independence, and multi-center studies establishing analytic validity are still absent.

The path forward is clear. Large international consortia should adopt harmonized high-density EEG protocols, preregistered pipelines that distinguish periodic from aperiodic activity and connectivity measures corrected for spatial leakage. Concurrent monitoring of cardiovascular and sleep variables should be standard. Linking electrophysiological data to genetic modifiers and real-world clinical outcomes will allow researchers to define prognostic thresholds and identify mechanistic targets for intervention. Advances in portable EEG technologies—such as dry-electrode or ear-EEG systems—coupled with privacy-preserving AI models will enable continuous, at-home monitoring at a global scale.

In parallel, the mechanistic insights gained—alpha loss as a readout of thalamocortical disinhibition, delta hypersynchrony as a marker of maladaptive gating, neurovascular uncoupling as an early systems-level deficit—open avenues for targeted intervention. Closed-loop tACS or DBS tuned to restore 7–9 Hz integrity, pharmacological enhancers of inhibitory tone (e.g., KCC2 modulators, astrocytic Kir4.1 up-regulation), and vascular-protective strategies can all be evaluated rapidly with electrophysiological endpoints.

In sum, EEG has progressed from a qualitative descriptor of “diffuse slowing” to a quantitative window on the circuit-level dynamics of HD. With coordinated standardization, longitudinal validation, and integration into adaptive-therapy trials, electrophysiology is poised to become a cornerstone of precision monitoring and network-based treatment of Huntington’s disease.

## Data Availability

No new data were created or analyzed in this study. Data sharing is not applicable to this article.
